# Comparison of koala LPCoLN and human strains of *Chlamydia pneumoniae *highlights extended genetic diversity in the species

**DOI:** 10.1186/1471-2164-11-442

**Published:** 2010-07-21

**Authors:** Candice M Mitchell, Kelley M Hovis, Patrik M Bavoil, Garry SA Myers, Jose A Carrasco, Peter Timms

**Affiliations:** 1 Institute of Health and Biomedical Innovation, Faculty of Science and Technology, Queensland University of Technology, Kelvin Grove, Queensland, 4059, Australia; 2 Department of Microbial Pathogenesis, University of Maryland, Baltimore, Maryland 21201, USA; 3 Institute for Genome Sciences, University of Maryland, Baltimore, Maryland 21201, USA

## Abstract

**Background:**

*Chlamydia pneumoniae *is a widespread pathogen causing upper and lower respiratory tract infections in addition to a range of other diseases in humans and animals. Previous whole genome analyses have focused on four essentially clonal (> 99% identity) *C. pneumoniae *human genomes (AR39, CWL029, J138 and TW183), providing relatively little insight into strain diversity and evolution of this species.

**Results:**

We performed individual gene-by-gene comparisons of the recently sequenced *C. pneumoniae *koala genome and four *C. pneumoniae *human genomes to identify species-specific genes, and more importantly, to gain an insight into the genetic diversity and evolution of the species. We selected genes dispersed throughout the chromosome, representing genes that were specific to *C. pneumoniae*, genes with a demonstrated role in chlamydial biology and/or pathogenicity (n = 49), genes encoding nucleotide salvage or amino acid biosynthesis proteins (n = 6), and extrachromosomal elements (9 plasmid and 2 bacteriophage genes).

**Conclusions:**

We have identified strain-specific differences and targets for detection of *C. pneumoniae *isolates from both human and animal origin. Such characterisation is necessary for an improved understanding of disease transmission and intervention.

## Background

The *Chlamydiaceae *are obligate intracellular pathogens that undergo a unique biphasic developmental cycle involving the inter-conversion between the extracellular infectious elementary body and the intracellular, replicative reticulate body. *Chlamydia 'Chlamydophila' pneumoniae *is probably one of the most successful chlamydial species, having established a niche in a range of warm-blooded (homoeothermic) and cold-blooded (poikilothermic) hosts, including humans, horses, marsupials, frogs and reptiles [1-5]. *C. pneumoniae *human infections are associated with bronchitis, pharyngitis, community-acquired pneumonia and more recently chronic diseases, such as atherosclerosis and stroke [6,7] myocarditis [8], multiple sclerosis [9] and Alzheimer's disease [10].

Australia's native icon, the koala (*Phascolarctos cinereus*), is found throughout Australia's north-eastern and southern eucalypt regions and is commonly infected with chlamydiae [11-14]. While the decline in koala populations has largely been the result of hunting and a diminished habitat, there is great concern for the koala due to an increased incidence of disease [15]. It is estimated that almost all of the nations free-range koala populations and many in captive populations are affected by *C. pneumoniae *and/or *C. pecorum *(the most common of the two species). *C. pneumoniae*-infected koalas may develop a respiratory illness similar to that in humans with clinical signs of sneezing, coughing, nasal discharge and chest congestion [16,17]. *C. pneumoniae *koala is not restricted to the respiratory tract and has been isolated from ocular and urogenital tract sites (often in conjunction with *C. pecorum*), although disease at these sites is poorly understood [13].

Exposure to *C. pneumoniae *is widespread due to effective aerosol transmission and outbreaks have been reported in humans [18-21], horses [22], frogs [23] and koalas [13]. However, the original source of infection remains undetermined. Myers *et al. *[24] recently published the full 1.24 Mbp genome sequence of the *C. pneumoniae *koala LPCoLN isolate, the first analysis of a *C. pneumoniae *genome from a non-human host species. This study revealed that the five *C. pneumoniae *genomes were highly similar in genomic organisation and gene order, although some notable differences were observed [24]. In contrast to the highly conserved human-derived isolates, a relatively high number of single nucleotide polymorphisms, SNPs (6213) differentiated koala LPCoLN from human AR39 [24]. In the proposed phylogeny (based on SNPs from 111 highly conserved genes), which encompassed all five *C. pneumoniae *genomes and five sequenced animal chlamydial genomes (*C. pecorum *E58, *C. muridarum *Nigg, *C. caviae *GPIC, *C. psittaci *6BC and *C. abortus *s26/3), koala LPCoLN was basal to the *C. pneumoniae *human isolates (larger genome and many full-length genes relative to human isolates).

In the present report we perform a comparative analysis of the whole koala LPCoLN genome sequence, highlighting the components that distinguish the strain isolated from the koala (animal), from those isolated from humans. These comparisons point to candidates of strain-specific adaptations and may provide potential targets for improved diagnostic tests, therapeutic intervention and epidemiological investigations.

## Results

We used a comparative genomics approach to identify genetic characteristics that were either unique to *C. pneumoniae *or were commonly shared with chlamydial species and other organisms. Overall, we analysed a total of 66 genes for key similarities and differences between human and animal strains of *C. pneumoniae *(see Additional file 1 for list of 66 genes analysed). We chose to use our *C. pneumoniae *animal genome as the reference genome and compared the available human genomes to it.

### *Chlamydia pneumoniae*-specific genes

Using tblastx and tblastn, we searched other genomes for orthologs of the genes identified from individual gene-by-gene comparisons of the five *C. pneumoniae *genomes. The comparative approach identified 140 genes that were specific to *C. pneumoniae *and for which no significant similarity was detected in any other organism (Additional file 2). Many of these species-specific genes are short open reading frames (ORFs) that have been annotated as genes. The large number of short hypothetical ORFs makes it difficult to determine whether these genes are 'real' or artefacts of the genome annotation or sequencing process. Until these proteins are systematically studied in the future, it cannot be determined whether these proteins are valid or too short to be protein-coding genes. Genes with suggested or predicted functions include putative lipoproteins and chlamydial inclusion membrane proteins (IncA). Several hypothetical proteins are clustered together (including CPK_ORF00340-343, 389-401, 496-498, 567-569, 658-661, 969-980: LPCoLN locus designation, CPK), suggesting that they may exist in an operon and might be functionally related.

### Genes with a demonstrated role in chlamydial biology and/or pathogenicity

One of the most striking differences between the *C. pneumoniae *koala and human genomes were changes associated with the polymorphic membrane proteins (Pmps). Like *C. pneumoniae *of human origin [25,26], koala LPCoLN is predicted to encode 21 Pmps that are phylogenetically related to one of six basic subtypes (*pmp*A, B/C, D, E/F, G/I and H; Additional files 3 and 4) [25,27-29]. The organisation of the *pmp *loci of koala LPCoLN is conserved relative to the *C. pneumoniae *human isolates. However, where the four human isolates carry several interrupted *pmp *genes (Additional file 3), koala LPCoLN carries uninterrupted, full-length versions of the same genes, including *pmp*G3, *pmp*G4 and *pmp*E3 (Additional file 3). A global comparison of all *pmp *sequences reveals a total of 2015 SNPs (of which 994 generated an amino acid change; see Additional file 5 for approximate SNP positions) differentiating koala LPCoLN from the four *C. pneumoniae *human isolates, with the highest percentage of SNPs observed in *pmp*E4 (30.46%) and *pmp*E3 (6.95%). In addition to SNPs, several strain-specific (human versus animal) indels (insertions and deletions) were evident in *pmp*B, *pmp*E1, *pmp*G2, *pmp*G4, *pmp*G5, *pmp*G7, *pmp*G10, and *pmp*G13 (Additional file 3). Interestingly, the *pmp*G5 pseudogene is interrupted by a stop codon in all five strains albeit at different sites: LPCoLN carries a seven nt indel (GAT GTA C) at nt position 332, resulting in a TAA stop codon at nt position 346, while the four human isolates have a SNP (C to T) at nt 1483, resulting in a TAA stop codon (data not shown). Previous analyses of human isolates have revealed variable numbers of 393 nt tandem repeat segments in *pmp*G6, including two repeats in AR39 [25] and J138 [30], and three repeats in TW183 (Geng MM, Schuhmacher A, Muehldorfer I, Bensch KW, Schaefer KP, Schneider S, Pohl T, Essig A, Marre R, Melchers K: The genome sequence of *Chlamydia pneumoniae *TW183 and comparison with other *Chlamydia *strains based on whole genome sequence analysis, submitted) and CWL029 [26]. The LPCoLN genome carries three variable tandem repeats in *pmp*G6.

Type III secretion (T3S) occurs independently of the *sec *pathway and requires assembly of a secretion apparatus composed of approximately 20 proteins. However, in this analysis we looked at more than just the apparatus proteins; we also examined potential secreted proteins and chaperone proteins involved in T3S. Additional file 6 compares 26 proteins from LPCoLN to putative orthologs of other chlamydial species. Ten apparatus-encoding genes (CDSs CPK_ORF00106, 111, 115, 231, 232, 233, 234, 236, 830 and 831), which were either annotated as such and/or found to be homologous to previously studied proteins in other chlamydial spp. were examined. Genetic comparisons indicate ≥ 98.2% nucleotide sequence identity between each koala LPCoLN T3S apparatus gene and orthologs from the human isolates. Similarly, comparisons of genes which were annotated as, or are similar to, chaperone-encoding genes that assist in the folding of effectors demonstrated high conservation with ≥ 99.3% sequence identity to the equivalent genes in the *C. pneumoniae *isolates from humans.

Nine putative effector-encoding genes (CDSs CPK_ORF00107, 216, 217, 430, 445, 446, 799, 800 and 832) of *C. pneumoniae *koala LPCoLN were compared to their counterparts in the human isolates. As with the T3S apparatus proteins, all koala LPCoLN effectors exhibited ≥ 98.2% sequence identity with their human isolate counterparts (Additional file 6).

**Figure 1:**
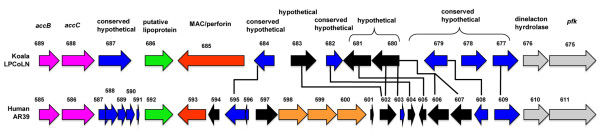
**Organisation of the *C. pneumoniae *plasticity zone**. A comparison of the *C. pneumoniae *koala LPCoLN and human AR39 genomes revealed evidence of fragmentation, gene decay, gene gain/loss in the plasticity zone. Genes are labelled with the published locus numbers. Lines connect orthologs. Role categories and colours are as follows: fatty acid and phospholipid metabolism, magenta; conserved hypothetical proteins, blue; cell envelope, light green; hypothetical proteins, black; biosynthesis of purines, pyrimidines, nucleosides, and nucleotides, orange; energy metabolism, light gray. Arrows indicate the direction of transcription.

The plasticity zone or replication termination region is a hypervariable region that is linked to genetic differences in chlamydial pathogen-host relationships. The membrane attack complex/perforin (MACPF) of the plasticity zone is one such protein that showed varied degrees of polymorphism among the chlamydial species. There was a significant length polymorphism differentiating the MACPF of *C. pneumoniae *koala (2457 nt CPK_ORF00685) from all four human isolates which encode a predicted defective MACPF separately incorporated into two ORFs, CP_0594 (381 nt) and CP_0593 (1236 nt) (Figure 1). A comparison with other chlamydial species showed that a *C. pneumoniae *ancestor separated from other '*Chlamydophila *spp.' before the large indel was removed from the human isolates. Similar to the *C. pneumoniae *koala LPCoLN MACPF, *C. trachomatis *serovar, A/HAR, B/Jali20/OT, D/UQ-3/CX, L2b/UCH-1/proctititis, L2/434/Bu and *C. muridarum *Nigg isolates had a full-length version (Additional file 7), while variations of the MACPF were observed in *C. abortus *S26/3, *C. caviae *GPIC and *C. felis *Fe/C-56 isolates (Additional file 7), which showed frame disruptions (likely pseudogenes). *Protochlamydia amoebophila *UWE25 did not have any detectable MACPF orthologs. MotifScans of the chlamydial MACPF (with the exception of *C. caviae, C. abortus *and *C. felis*) revealed an MIR (Mannosyltransferase, Inositol 1,4,5-trisphosphate receptor and Ryanodine receptor) motif, suggestive of a possible ligand transferase function. The size variation of the *C. pneumoniae *MACPF may serve as a useful marker in future genetic investigation. For example, the MACPF gene sequence may potentially differentiate *C. pneumoniae *animal isolates from *C. pneumoniae *human isolates.

### Genes involved in nucleotide salvaging pathways or amino acid biosynthesis

All sequenced chlamydial genomes to date encode a CTP synthetase, the enzyme that converts UTP to CTP, and an ATP/ADP translocase [25,31]. However, comparative genomic analysis suggests that multiple modifications have occurred in nucleotide salvage pathways during the course of chlamydial evolution, as revealed by the variable presence of *udk *(uridine kinase), *pyrE *(pyrimidine phosphoribosyl transferase), *guaB *(IMP dehydrogenase), *guaA *(GMP synthase) and *add *(adenosine deaminase) in different isolates (Table 1).

**Table 1:** *Chlamydiaceae *genome features with suspected host and niche specific genes

Species	Genome size (nt)	Protein coding sequences	Tryptophan metabolism	Toxin genes	Plasmid	Bacteriophage	*tyr*P copies	Nucleotide salvaging
*C. pneumoniae *AR39	1229853	1052	*tph*	Absent	Absent	Present	Two	*guaBA*-*add, udk, pyrE*
*C. pneumoniae *CWL029	1230230	1073	*tph*	Absent	Absent	Absent	Two	*guaBA*-*add, udk, pyrE*
*C. pneumoniae *J138	1226565	1072	*tph*	Absent	Absent	Absent	One	*guaBA*-*add, udk, pyrE*
*C. pneumoniae *TW183	1225935	1113	*tph*	Absent	Absent	Absent	One	*guaBA*-*add, udk, pyrE*
*C. pneumoniae *LPCoLN	1241024	1095	*tph*	Absent	Present	Present*	One	*udk, pyrE*
*C. felis *Fe/C-56	1166239	1005	*trpABFCDR, kynU*	Absent	Present	Absent	One	*guaBA*-*add, pyrE*
*C. caviae *GPIC	1173390	1009	*trpABFCDR, kynU, prsA, tph*	Present	Present	Present	One	*guaBA*-*add, pyrE*
*C. abortus *S26/3	1144377	961	*tph*	Absent	Absent	Present	One	*guaB *(pseudogene), *pyrE*
*C. muridarum *Nigg	1069412	924	none	Present	Present	Absent	Two	*guaBA*-*add, upp*
*C. trachomatis *serovar D	1042519	894	*trpABCR*	Present	Present	Absent	Two	None of the above

*tph*, tryptophan hydroxylase; *trp*, tryptophan biosynthesis; *kynU*, kynureninase; *prsA*, ribose-phosphate pyrophosphokinase; *gua*, purine biosynthesis; *add*, adenosine deaminase; *udk*, uridine kinase; *pyrE*, UMP synthase; *upp*, uracil phosphororibosyl transferase. * Asterisk indicates remnants.

*C. pneumoniae *is the only chlamydial species to have a *udk *gene for UMP production. Our examination of the *C. pneumoniae *sequence revealed that the 3' end of the gene was unique to the species. No significant sequence similarity was observed in other organisms between nt regions 541-669, indicating that this region might be specific for *C. pneumoniae*. A sequence alignment of the full-length *udk *gene identified only three SNPs (one amino acid change) differentiating koala LPCoLN from the sequenced human isolates.

The bacterial pyrimidine biosynthesis pathway includes several enzymes for the conversion of UMP into CTP. However, all chlamydial genomes thus far lack the genes for most of the pathway with the exception of the last few steps (Additional file 8). *C. pneumoniae *of koala and human origins, *C. felis, C. caviae *and *C. abortus *have the *pyrE *gene, encoding an orotate phosphoribosyl transferase involved in pyrimidine biosynthesis. However, the final step in *de novo *pyrimidine biosynthesis is via orotidine-5'-monophosphate decarboxylase (*pyrF*) and this gene is missing from all chlamydial genomes.

The purine biosynthesis pathway is also incomplete, similar to the pyrimidine pathway, with many genes variably missing in the chlamydial genomes (Table 1). The only four genes that are absent from the koala LPCoLN genome but are present in the human genomes include CP_0597, which encodes a hypothetical protein, *guaB *(IMP dehydrogenase), *guaA *(GMP synthase) and *add *(AMP adenosine deaminase), which are involved in purine ribonucleotide biosynthesis. The *guaA *and *add *sequences of the *C. pneumoniae *human isolates were identical, while *guaB *fragmentation was evident in TW183, CWL029 and J138 isolates with a deleted 324 nt at the 5' end of the sequence (Figure 1). The CP_0597 gene resides next to this *guaBA*-*add *cluster (Figure 1), which may indicate that this gene may also be involved in purine biosynthesis.

The tryptophan biosynthesis operon is missing from several chlamydial species including *C. muridarum *Nigg, *C. abortus *S26/3, and *C. pneumoniae *of both koala and human origin (Table 1). Despite this absence, *C. pneumoniae *koala and human encode a functional aromatic amino acid (tryptophan) hydroxylase, although the koala LPCoLN isolate is missing the extended N-terminal region [32]. While variations in *C. pneumoniae tyr*P (tryptophan tyrosine permease) copy numbers have been found between human isolates [33], we report that koala LPCoLN, a respiratory isolate, has a single copy of *tyr*P*. *A comparison of the *tyr*P sequence from all five sequenced (full-genome) *C. pneumoniae *isolates revealed that the sequence was highly conserved across the seven copies of *tyr*P, revealing only nine SNPs including seven unique to koala LPCoLN, four of which led to an amino acid change. Previously, Gieffers *et al. *[33] published a *tyr*P-specific SNP profile of 20 *C. pneumoniae *human isolates, and here we report the SNP profile of two additional respiratory isolates J138 (CGGGG) and LPCoLN (CAAGG).

### Extrachromosomal elements of the *Chlamydiaceae*

**Figure 2:**
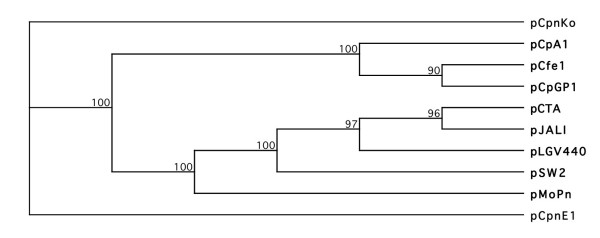
**Phylogeny of the chlamydial plasmid**. Phylogenetic relationships of *C. pneumoniae *koala LPCoLN (pCpnKo), *C. pneumoniae *horse N16 (pCpnE1), *C. psittaci *avian N352 (pCpA1), *C. felis *feline Fe/C-56 (pCfe1), *C. caviae *guinea pig GPIC (pCpGP1), *C. muridarum *mouse Nigg (pMoPn) and *C. trachomatis *human serovars A (pCTA), B (pJALI), E (pSW2) and L1 (pLVG440) were inferred from predicted amino acid sequences, and were constructed by Neighbor-Joining analysis and 1,000 bootstrap replicates.

Extrachromosomal plasmid sequences pCpnKo from *C. pneumoniae *koala LPCoLN [24], pCpnE1 from *C. pneumoniae *horse N16 [34], pCpA1 from *C. psittaci *avian N352 (Lusher ME, Gregory J, Storey CC, Richmond SJ: Analysis of the complete nucleotide sequence of the plasmid pCpA1 isolated from an avian strain of *Chlamydia psittaci*, Submitted), pCfe1 from *C. felis *feline Fe/C-56 [35], pCpGP1 from *C. caviae *guinea pig GPIC [36], pMoPn from *C. muridarum *mouse Nigg [25], and pCTA, pJALI, pSW2 and pLVG440 from *C. trachomatis *human serovars A [37], B [38], E [38] and L1 [39], respectively, were compared for their overall synteny and relationship (Additional file 9). These plasmids each contain eight major open reading frames (ORFs – potentially encode a protein) designated ORF1-8. Although the plasmid sequences vary in size (7169-7966 bp), alignment of their amino acid sequences revealed a high degree of similarity and large conserved regions (Additional files 9). The pCpnKo and pCpnE1 plasmid sequences share 96.2% identity and are more closely related to pCpA1 (81.3 and 78.9% identity), pCpGP1 (81.6 and 79.1% identity) and pCfe1 (77.5 and 75.1% identity) than with pMoPn (69.5 and 67.7% identity), pCTA, pJALI, pSW2 and pLVG440 (63.9-69.5% identity) (Additional file 10). Overall, there were approximately 30 indels among the species; three of the longest indels were identified in ORF1 of pCpnE1 (deletion), pCfe1 (insertion) and pSW2 (deletion). A phylogenetic tree was inferred from multiple sequence alignment of the amino acid sequence (Figure 2). Three main branches (supported with a high bootstrap value) were evident: (1) *C. pneumoniae *LPCoLN and N16; (2) *C. psittaci *N352, *C. felis *Fe/C-56 and *C. caviae *GPIC; (3) *C. muridarum *and *C. trachomatis *isolates.

The bacteriophage is another strain-specific extrachromosomal element reported in chlamydial species. In *C. pneumoniae*, AR39 is the only human genome to have an extrachromosomal bacteriophage (4524 nt single-stranded DNA) [25], whereas the koala LPCoLN genome showed remnants of a phage. The first remnant of a phage in the koala LPCoLN genome was evident in CPK_ORF00729, a 366 nt incomplete ORF that is presumably defective, sharing approximately 79% similarity (nt 301/382) to the partial-length of the human AR39 phage. CPK_ORF00729 also shares 97% similarity (nt 324/333) to the Chp1 remnant (*C. psittaci *phage), which is present in the four *C. pneumoniae *human genomes. This suggests earlier integration of the phage genome in the *C. pneumoniae *genome (Geng MM, Schuhmacher A, Muehldorfer I, Bensch KW, Schaefer KP, Schneider S, Pohl T, Essig A, Marre R, Melchers K: The genome sequence of *Chlamydia pneumoniae *TW183 and comparison with other *Chlamydia *strains based on whole genome sequence analysis, submitted). The second koala LPCoLN phage remnant was a 445 nt ORF, termed CPK_ORF00730, which appears to be 'intact', sharing approximately 77% similarity (nt 342/445) to the human AR39 phage. The koala LPCoLN phage remnants are positioned between hypothetical genes in the genome and there appear to be no further remnants of the phage within this region of the genome. A comparison of the *C. pneumoniae *koala LPCoLN phage with other chlamydial species revealed approximately 77-79% sequence identity to partial-length sequences of the *C. psittaci *Chp2 phage, *C. pecorum *phage 3, *C. caviae *phiCPG1 phage and *C. abortus *Chp4 phage.

## Discussion

In this work, we applied computational analyses to explore the genome content and genetic diversity among the recently sequenced *C. pneumoniae *koala LPCoLN genome and previously published *C. pneumoniae *human genomes (AR39, CWL029, J138 and TW183). The koala LPCoLN genome is larger than all four *C. pneumoniae *human genomes by 10-12 kbp. We combined BLAST search methods and motif analysis for use in elucidating the relationship between gene function and evolution. Even though these techniques have several limitations [40], our comparative approach has (i) identified genome plasticity, (ii) provided circumstantial evidence for the presumed direction of *C. pneumoniae *evolution, and (iii) suggested targets for detection and differentiation of *C. pneumoniae *isolates from both human and animal origins.

The presence of unique insertions/deletions is evidence of evolution in action. For the majority of these genes, the *C. pneumoniae *koala genome has the full-length version. These length polymorphisms suggest that the presumed functional changes are brought about by adaptation to a specialised niche, where the ancestral gene function may no longer be required. Therefore, it is important to understand how these genetic differences may influence differences in pathogenicity and fitness in the host. Our data supports the findings of Rattei *et al. *[41] and the whole genome findings of Myers *et al. *[24] in that the essentially clonal human isolates have evolved from an animal strain(s) that has adapted to humans through fragmentation, decay and loss-of-function processes whereby the activity of the gene product may be reduced or specialised. Hence, the koala LPCoLN genome seems to be an 'older' strain in this sense. In addition, we provide new information on strain diversity and have identified targets for detection and further investigation.

Our analysis revealed a total of 140 genes that were specific to *C. pneumoniae*. One hundred and twenty-three of these represented hypothetical genes with no significant similarity to genes in other organisms present in the database. Further analysis of these hypothetical genes (subcellular localisation, gene expression analysis, functional profiling from microarray analysis) may reveal undiscovered biovars or subspecies in *C. pneumoniae*.

The Pmp family is characterised by an unusual degree of sequence polymorphism, including mutations and large indels across all species [42-47] and showed variation within *C. penumoniae*. This suggests that the *pmp *gene family is subjected to high selective pressure (niche, host-specific or immune-mediated), correlating with a relatively faster evolutionary rate for these antigens. Taken together, the polymorphism of *pmp *sequences in *C. pneumoniae *from humans and animals is dually consistent with the divergent evolution of the *pmp *genes under host-specific selection while maintaining the capacity to adapt to specific niches or immune responses in the two different hosts.

In light of the variation seen between other families of genes and their orthologs in the human isolates, namely the *pmp *protein family, the strict conservation of T3S effector genes was initially surprising given the effectors' normal tendency for divergence. While the differences observed between other regions of the genomes are consistent with evolutionary changes [24], the relative conservation of effector genes over equivalent time suggest that changes in genes encoding effector proteins were likely selected against. This is consistent with a key role of T3S effectors in mediating steps of the biology of *C. pneumoniae *that are conserved in human and animal strains, such as inclusion and intracellular development. Overall, these results support a critical role for T3S less in the virulence than in the developmental biology of these organisms. Such a role has been proposed in the context of the contact-dependent T3S-mediated hypothesis of chlamydial development proposed earlier [48,49].

Orthologs of the MACPF were identified in several chlamydial species. The first biological characterisation of the *C. trachomatis *MACPF by Taylor *et al. *[50] has revealed that the MACPF (CT153) might be activated by proteolytic processing and may play a role in the acquisition or modification of host-derived lipids. By contrast, studies of the MACPF in other organisms, including that of *Toxoplasma *spp. have shown that ablation of the MACPF (termed TgPLP1) resulted in a reduction in virulence (in mice), whereby TgPLP1 deficient parasites were unable to exit normally and were entrapped within host cells, due to the inability to permeabilise the parasitophorous vacuole membrane [51]. If the chlamydial MACPF was to play a similar role in egression or virulence, then why have several species failed to retain this gene? Non-lytic family members have also been identified in other organisms including Astrotactin involved in neural migration in mammals [52], a *Drosophila *torso-like protein involved in embryonic development [53] and Plu-MACPF of *Photorhabdus luminescens *which binds to the surface of insect cells [54]. Further investigation of this gene should provide more insight into its role in *Chlamydiaceae.*

*C. pneumoniae *is the only chlamydial species thus far to have a *udk *gene encoding uridine kinase. The *udk *gene is a pyrimidine ribonucleoside kinase that phosphorylates uridine and cytidine into uridine or cytidine monophosphate (UMP/CMP) [55], and is highly conserved in the species. It has been reported that the *Prevotella bryantii *genome encodes a putative uracil DNA glycosylase and uridine kinase, likely to be involved in the removal of misincorporated uracil from DNA and its subsequent re-use [56]. All chlamydial genomes also encode a uracil DNA glycosylase, however, *C. pneumoniae *is the only species carrying the *udk *ortholog. This implies that an alternative gene product is involved in UMP production in the other chlamydial species. The *C. muridarum *genome includes a CDS (*upp*) encoding a uracil phosphoribosyltransferase [25] that may represent the main pathway for UMP production in this species. *C. pneumoniae *is unusual in having a very broad host range and therefore the fact that it is the only chlamydial species to have retained the *udk *gene could reflect this broad host capacity.

Most bacteria can salvage or synthesise their own purines and pyrimidines. By contrast, chlamydiae and rickettsiae (another obligate intracellular bacterium) are incapable of *de novo *synthesis, and to a degree, of salvage [31,57]. Given the absence of genes for enzymes upstream in the pyrimidine biosynthesis pathway, it is unclear why the *pyrE *should be retained. The final step in the pathway is via *pyrF *which is absent from the chlamydial genome, suggesting that they are unable to convert orotate to UMP. The presence in all chlamydial genomes of orthologs encoding the three downstream enzymes involved in UMP to CTP conversion and the earlier demonstration of CTP synthetase activity in these organisms [58,59] confirm that chlamydiae are not auxotrophic for CTP. Furthermore, a three-gene cluster including *guaB*, *guaA *and *add *have been selectively maintained in several chlamydial species including *C. pneumoniae *AR39, CWL029, TW183 and J138, *C. felis *Fe/C-56, *C. caviae *GPIC and *C. muridarum *Nigg. *C. abortus *has a *guaB *pseudogene, whereas the *C. pneumoniae *LPCoLN and *C. trachomatis *serovar A/HAR, B/Jali20/OT, D/UQ-3/CX, L2b/UCH-1/proctititis, L2/434/Bu and *Candidatus Protochlamydia amoebophila *UWE25 genomes lack all three genes [25,26,30,35,36,60]. The selective loss of *guaBA*-*add *from *C. pneumoniae *koala LPCoLN and other chlamydial species suggest that these three enzymes required for inter-conversion of GMP, IMP and AMP must be acquired by other means or are clearly not essential for species survival.

Copy number variations of the *tyr*P (tryptophan tyrosine permease) gene have been suggested to reflect vascular tropism and pathogenicity among *C. pneumoniae *human isolates with multiple copies associated with respiratory infection and single copy more frequently associated with vascular tropism [33]. A comparison of the five sequenced *C. pneumoniae *genomes also revealed variations in the *tyr*P (tryptophan tyrosine permease) copy number that are, however, inconsistent with the hypothesis by Gieffers *et al. *[33]. Among these, two respiratory isolates (koala LPCoLN and human J138) [24,30], as well as the single conjunctival isolate (TW183) of the group (Geng MM, Schuhmacher A, Muehldorfer I, Bensch KW, Schaefer KP, Schneider S, Pohl T, Essig A, Marre R, Melchers K: The genome sequence of *Chlamydia pneumoniae *TW183 and comparison with other *Chlamydia *strains based on whole genome sequence analysis, submitted) have a single *tyr*P copy while two other respiratory isolates (CWL029 and AR39) [25,26] carry duplicate copies of *tyr*P.

The loss and fragmentation of pre-existing genes during evolution is one of the primary distinguishing features between *C. pneumoniae *koala and *C. pneumoniae *human. Extrachromosomal plasmids have been identified in six of the nine chlamydial species. As the plasmid is not common to *C. pneumoniae*, it is not known why koala LPCoLN has a plasmid.

While proteins with predicted or known biologic function are favoured candidate gene targets, many *C. pneumoniae*-specific hypothetical proteins with no predicted function were identified in the comparisons and may be worth further investigating for their potential role in host tropism, pathogenicity and niche adaptation (see Additional file 2 for the list of genes). A suggested list of target genes for *C. pneumoniae *detection and a brief description of their characteristics is summarised in additional file 11. Selected genes include (i) *C. pneumoniae*-specific genes for detection of *C. pneumoniae*, (ii) genes that could potentially differentiate isolates from human and animal origins, for example, length polymorphic genes including the membrane attack complex perforin and the hypothetical protein CPK_ORF00679, (iii) genes for the identification of a *C. pneumoniae *plasmid.

## Conclusions

The study of whole genome sequences provides important clues to the natural history of *C. pneumoniae *and their hosts, and assists in the identification of functional differences that may determine pathogenicity and virulence differences between the strains. Previously, whole genome comparisons have focused on four practically clonal *C. pneumoniae *human genomes, which make the identification of target genes for strain differentiation quite challenging. In this study we have made use of the recently sequenced koala LPCoLN genome, which is the largest and most unique *C. pneumoniae *genome sequenced thus far, in order to select target genes that represented traits of strain-specific determinants. Further investigation of these genes in other host species may provide additional clues as to what enables this pathogen to: (i) present varied clinical pathologies, (ii) occupy multiple niches, and (iii) establish an infection in cold and warm-blooded hosts. Moreover, these genes may become targets for improved diagnosis and therapeutic strategies.

## Methods

### *Chlamydia pneumoniae *isolates

The complete genomic sequences of *C. pneumoniae *AR39 (GenBank accession number AE002161), CWL029 (GenBank accession number AE001363) TW183 (GenBank accession number AE009440), J138 (GenBank accession number BA000008) and LPCoLN (GenBank accession CP001713) were used in this study.

### Strategy for selection of genes used for comparisons

The selection strategy involved individual gene-by-gene comparisons of the complete koala LPCoLN genome. NCBI BLAST [61] searches using tblastx and tblastn were conducted in order to search for orthologs in the *C. pneumoniae *human genome and other organisms using an E-value cutoff of 1 × 10^-4 ^with manual curation. This approach enabled us to identify regions of high SNP accumulation and to select target genes for comparison. These selected genes were grouped into five categories by their putative, predicted or hypothetical functions. The categories included: (1) C. pneumoniae-specific genes (with respect to the koala LPCoLN genome, n = 140); (2) genes with a prior demonstrated role in chlamydial biology and pathogenicity (n = 49); (3) genes encoding nucleotide salvage or amino acid biosynthesis proteins (n = 6); (4) extrachromosomal elements, including a plasmid (n = 9) and bacteriophage-related genes (n = 2) (Additional files 1 and 2). Selected genes were individually aligned in order to identify SNPs, indels and targets for strain-specific adaptations.

### Analysis of nucleotide and amino acid sequences and phylogeny

Gene alignments were performed using the Clustal W program (MacVector 10.6 Genetics Computer Group, Madison, Wisconcin) to identify polymorphisms and indels (insertions/deletions) [62]. Conserved residues are outlined, similar amino acids are shaded in grey, mismatches are not shaded and dashes correspond to gaps in the sequence. A consensus line appears at the bottom of the alignment. Pmp sequences were subjected to multiple sequence alignment using ClustalW2 (EMBL-EBI) [63] and BioEdit Sequence Alignment Editor [64]. SNP position analysis for each group was performed using the Microsoft Excel program (Microsoft Corporation). Motif Scan [65] was used to identify motifs in a sequence.

A phylogenetic tree was constructed by Neighbor-Joining, tie breaking = systematic, distance corrected by the Poisson method with gaps distributed proportionally and 1,000 bootstrap replicates.

## Abbreviations

CDS: Coding Sequence; Indels: Insertions/Deletions; ORF: Open Reading Frame; SNP: Single Nucleotide Polymorphism.

## Authors' contributions

CMM prepared the manuscript and performed comparative analyses including evaluations. KMH contributed with text, interpretation and analysis for the manuscript. PMB aided in data interpretation and critically revised the manuscript. GSAM interpretation and analysis of the genome. JAC contributed with text, interpretation and analysis for the manuscript. PT participated in the design and drafting of the manuscript, aided in data interpretation and critically revised the manuscript. All authors read and approved the final manuscript.

## Supplementary Material

Additional file 1 **Categories of targets for investigation.** (1) genes with a prior demonstrated role in chlamydial biology and pathogenicity (n = 49); (2) genes encoding nucleotide salvage or amino acid biosynthesis proteins (n = 6); (3) extrachromosomal elements, including a plasmid (n = 9) and bacteriophage-related genes (n = 2). Click here for file

Additional file 2 ***C. pneumoniae*-specific genes**. A list of *C. pneumoniae *genes that have no significant similarity to other chlamydial species or organisms. E-value cutoff of 1 × 10^-4 ^with manual curation. Click here for file

Additional file 3 **Comparative analysis of the *C. pneumoniae *polymorphic membrane proteins (Pmps)**. A comparison of the 21 Pmps revealed a high degree of sequence polymorphism and indels between the koala LPCoLN and human AR39, CWL029 and TW183 isolates. The TW183 and J138 isolates were well-conserved and therefore, TW183 also represents J138 in this figure. Arrows indicate the direction of transcription: green arrows show typical *pmp *characteristics, red arrows represent pseudogenes (numbers below arrows indicate stop codon position), and white arrows represent proteins with no relation to *pmps *(note the same orientation as *pmp*G10). Dashed lines indicate truncated products. Click here for file

Additional file 4 **Polymorphic outer membrane protein features of *C. pneumoniae***. Click here for file

Additional file 5 **Polymorphic outer membrane protein SNP position analysis of *C. pneumoniae***. Click here for file

Additional file 6 **T3S ortholog comparisons**. Click here for file

Additional file 7 ***Chlamydia *MACPF**. A BLAST alignment of the *C. pneumoniae *MACPF protein. From top to bottom: *C. pneumoniae *LPCoLN, *C. pneumoniae *J138, *C. pneumoniae *CWL029, *C. felis *FE/C-56, *C. trachomatis *A/HAR-13, *C. trachomatis *6276, *C. trachomatis *D/UW-3/CX, *C. trachomatis *70, *C. trachomatis *434/Bu, *C. muridarum *Nigg, *C. pneumoniae *CWL029, *C. abortus *S26/3, *C. felis *Fe/C-56, *Alcanivorax *sp. DG881, *Saccolglossus kowaleski*, *Theileria parva *strain Muguga.... Click here for file

Additional file 8 ***Chlamydia *has lost several steps in the pyrimidine biosynthesis pathway**. All chlamydial genomes sequenced thus far, have lost the initial steps involved in pyrimidine biosynthesis. *C. pneumoniae *(Cpn), *C. abortus *(Cab), *C. caviae *(Cav) and *C. felis *(Cfe) contain a *pyrE *gene encoding an orotate phosphoribosyltransferase, while *C. muridarum *(Cmu) and *C. trachomatis *(Ctr) lack this gene. The next step in the pathway is via *pyrF*, which is absent from all chlamydial genomes. Interestingly, all six genomes have maintained the last three steps for the conversion of UMP into CTP. Adapted from Koonin and Galperin [66]. Gene names: *carA*, carbamoyl-phosphate synthase, small subunit; *carB*, carbamoyl-phosphate synthase, large subunit; *pyrB*, aspartate carbamoyltransferase; *pyrC*, dihydroorotase; *pyrD*, dihydroorotate dehydrogenase; *pyrE*, orotate phosphoribosyltransferase; *pyrF*, orotidine 5-phosphate decarboxylase; *pyrH*, uridylate kinase; *ndk*, nucleoside diphosphate kinase; *pyrG*, CTP synthase. Red boxes indicate gene loss, yellow boxes indicate the presence of a gene. Click here for file

Additional file 9 **Sequence comparison of the chlamydial plasmid**. Multiple sequence alignment of the predicted amino acid sequence from *C. pneumoniae *koala LPCoLN (pCpnKo), *C. pneumoniae *horse N16 (pCpnE1), *C. psittaci *avian N352 (pCpA1), *C. felis *feline Fe/C-56 (pCfe1), *C. caviae *guinea pig GPIC (pCpGP1), *C. muridarum *mouse Nigg (pMoPn) and *C. trachomatis *human serovars A (pCTA), B (pJALI), E (pSW2) and L1 (pLVG440). The sequences are well-conserved across species, indicating some degree of ancestry among them. The *C. pneumoniae *plasmid shared a close relationship with *C. psittaci, C. caviae *and *C. felis*, while *C. muridarum *and *C. trachomatis *were highly conserved. Predicted functions include plasmid replication (ORF1 and ORF2), double-stranded DNA unwinding (ORF3), chlamydial pathogenesis (ORF5) and regulation of partitioning and copy number (ORF7 and ORF8) [34,67]. The functions of ORF4 and ORF6 remain to be determined. Click here for file

Additional file 10 **Plasmid similarity scores (%)**. Plasmid similarity scores based on multiple sequence alignment. Click here for file

Additional file 11 **List of *C. pneumoniae *target genes**. Suggested target genes for detection, strain differentiation and plasmid identification in *C. pneumoniae. *See also reference [68]. Click here for file
