# Supplementary material for: Comparison of koala LPCoLN and human strains of Chlamydia pneumoniae highlights extended genetic diversity in the species
Source: BMC Genomics. 2010 Jul 21;11:442. doi: 10.1186/1471-2164-11-442 (PMC3091639; doi:10.1186/1471-2164-11-442)
Supplement: Additional file 2 — C. pneumoniae-specific genes. A list of C. pneumoniae genes that have no significant similarity to other chlamydial species or organisms. E-value cutoff of 1 × 10^-4^ with manual curation. [file 1471-2164-11-442-S2.DOC]

## Additional file 2 - *C. pneumoniae*-specific genes

| **Koala LPCoLN**  **locus designation** | **ORF size (aa)** | **Human AR39**  **locus designation** | **ORF size (aa)** | **Predicted or known function** |
| --- | --- | --- | --- | --- |
| CPK_ORF00015 | 43 |  |  | Hypothetical |
| CPK_ORF00064 | 67 | CP_0083 | 67 | Hypothetical |
| CPK_ORF00079 | 47 |  |  | Putative lipoprotein |
| CPK_ORF00087 | 37 | CP_0061 | 37 | Hypothetical |
| CPK_ORF000119 | 58 |  |  | Hypothetical |
| CPK_ORF000193 | 40 |  |  | Hypothetical |
| CPK_ORF00206 | 93 | CP_1072 | 349 | Hypothetical |
| CPK_ORF00237 | 201* | CP_1042 | 185 | Hypothetical |
| CPK_ORF00288 | 53 |  |  | Hypothetical |
| CPK_ORF00295 | 53 |  |  | Hypothetical |
| CPK_ORF00297 | 68 |  |  | Hypothetical |
| CPK_ORF00304 | 37 |  |  | Hypothetical |
| CPK_ORF00340 | 49 | CP_0936 | 45 | Hypothetical |
| CPK_ORF00341 | 384 | CP_0935 | 158 | Hypothetical |
| CPK_ORF00343 | 42 | CP_0933 | 46 | Hypothetical |
| CPK_ORF00346 | 45 | CP_0930 | 45 | Hypothetical |
| CPK_ORF00357 | 40 | CP_0916 | 40 | Hypothetical |
| CPK_ORF00361 | 57 |  |  | Hypothetical |
| CPK_ORF00389 | 45 |  |  | Hypothetical |
| CPK_ORF00390 | 38 |  |  | Hypothetical |
| CPK_ORF00391 | 40 |  |  | Hypothetical |
| CPK_ORF00392 | 346 | CP_0879 | 343 | Hypothetical |
| CPK_ORF00397 | 37 | CP_0884 | 44 | Hypothetical |
| CPK_ORF00398 | 44 | CP_0884 | 44 | Hypothetical |
| CPK_ORF00399 | 80 | CP_0883 | 44 | Hypothetical |
| CPK_ORF00400 | 207 | CP_0882 | 164 | Hypothetical |
| CPK_ORF00401 | 370 | CP_0880 | 367 | Hypothetical |
| CPK_ORF00402 | 343 | CP_0879 | 343 | Hypothetical |
| CPK_ORF00403 | 343 | CP_0878 | 343 | Hypothetical |
| CPK_ORF00479 | 771 | CP_0764 | 774 | IncA family protein |
| CPK_ORF00489 | 56 | CP_0785 | 56 | Hypothetical |
| CPK_ORF00496 | 38 |  |  | Hypothetical |
| CPK_ORF00497 | 81 | CP_0779 | 480 | Hypothetical |
| CPK_ORF00498 | 60 | CP_0779 | 480 | Hypothetical |
| CPK_ORF00502 | 50 | CP_0774 | 50 | Hypothetical |
| CPK_ORF00507 | 46 |  |  | Hypothetical |
| CPK_ORF00510 | 755 | CP_0765/766 | 435/267 | IncA family protein |
| CPK_ORF00511 | 275 | CP_0733 | 449 | IncA family protein |
| CPK_ORF00513 | 774 | CP_0762/763 | 523/241 | IncA family protein |
| CPK_ORF00517 | 62 | CP_0758 | 62 | Putative lipoprotein |
| CPK_ORF00520 | 37 |  |  | Hypothetical |
| CPK_ORF00527 | 288 | CP_0750 | 288 | Hypothetical |
| CPK_ORF00529 | 38 |  |  | Hypothetical |
| CPK_ORF00532 | 41 |  |  | Hypothetical |
| CPK_ORF00545 | 39 |  |  | Hypothetical |
| CPK_ORF00546 | 772 | CP_0764 | 774 | IncA family protein |
| CPK_ORF00548 | 756 | CP_0728 | 735 | IncA family protein |
| CPK_ORF00549 | 735 | CP_0728 | 735 | IncA family protein |
| CPK_ORF00550 | 66 | CP_0727 | 66 | Hypothetical |
| CPK_ORF00552 | 157 | CP_0725 | 160 | Hypothetical |
| CPK_ORF00553 | 162 |  |  | Hypothetical |
| CPK_ORF00555 | 39 |  |  | Hypothetical |
| CPK_ORF00560 | 38 |  |  | Hypothetical |
| CPK_ORF00567 | 38 | CP_0712 | 38 | Hypothetical |
| CPK_ORF00568 | 52 | CP_0711 | 52 | Hypothetical |
| CPK_ORF00569 | 37 |  |  | Hypothetical |
| CPK_ORF00573 | 367 | CP_0707 | 367 | Hypothetical |
| CPK_ORF00575 | 38 |  |  | Hypothetical |
| CPK_ORF00577 | 42 |  |  | Hypothetical |
| CPK_ORF00584 | 41 |  |  | Hypothetical |
| CPK_ORF00618 | 37 |  |  | Hypothetical |
| CPK_ORF00622 | 37 | CP_0663 | 37 | Hypothetical |
| CPK_ORF00637 | 170 | CP_0648 | 170 | Hypothetical |
| CPK_ORF00638 | 797 | CP_0647/646 | 56/759 | IncA family protein |
| CPK_ORF00643 | 344 | CP_0641 | 346 | Hypothetical |
| CPK_ORF00658 | 39 |  |  | Hypothetical |
| CPK_ORF00659 | 142 | CP_0627 | 167 | Hypothetical |
| CPK_ORF00660 | 63 |  |  | Hypothetical |
| CPK_ORF00661 | 123 | CP_0626 | 149 | Hypothetical |
| CPK_ORF00666 | 39 | CP_0621 | 39 | Hypothetical |
| CPK_ORF00668 | 40 | CP_0619 | 40 | Hypothetical |
| CPK_ORF00671 | 66 | CP_0616 | 68 | Hypothetical |
| CPK_ORF00672 | 40 |  |  | Hypothetical |
| CPK_ORF00674 | 332 | CP_0613/612 | 179/102 | Hypothetical |
| CPK_ORF00677 | 342 | CP_0609 | 342 | Hypothetical |
| CPK_ORF00680 | 262 | CP_0607/606 | 185/195 | Hypothetical |
| CPK_ORF00681 | 369 | CP_0605/606/607 | 111/195/185 | Hypothetical |
| CPK_ORF00683 | 365 | CP_0602 | 264 | Hypothetical |
| CPK_ORF00710 | 65 | CP_0562 | 65 | Hypothetical |
| CPK_ORF00714 | 325 | CP_0557 | 81 | Hypothetical |
| CPK_ORF00715 | 333 | CP_0557 | 81 | Hypothetical |
| CPK_ORF00716 | 439 | CP_0554/555 | 98/315 | IncA family protein |
| CPK_ORF00717 | 393 | CP_0553 | 393 | IncA family protein |
| CPK_ORF00718 | 43 | CP_0552 | 43 | Hypothetical |
| CPK_ORF00719 | 435 | CP_0551 | 404 | IncA family protein |
| CPK_ORF00720 | 419 | CP_0550 | 419 | IncA family protein |
| CPK_ORF00721 | 145 | CP_0549 | 145 | Hypothetical |
| CPK_ORF00722 | 50 |  |  | Hypothetical |
| CPK_ORF00728 | 38 |  |  | Hypothetical |
| CPK_ORF00731 | 38 |  |  | Hypothetical |
| CPK_ORF00735 | 223 | CP_0539 | 223 | IncA family protein |
| CPK_ORF00749 | 395 | CP_0522 | 388 | Hypothetical |
| CPK_ORF00751 | 144 | CP_0520 | 144 | Hypothetical |
| CPK_ORF00752 | 47 |  |  | Hypothetical |
| CPK_ORF00753 | 37 |  |  | Hypothetical |
| CPK_ORF00754 | 140 | CP_0519 | 141 | Hypothetical |
| CPK_ORF00785 | 49 | CP_0482 | 49 | Hypothetical |
| CPK_ORF00786 | 169 | CP_0481 | 169 | Hypothetical |
| CPK_ORF00791 | 554 | CP_0474/475 | 165/969* | Hypothetical |
| CPK_ORF00834 | 39 |  |  | Hypothetical |
| CPK_ORF00836 | 39 | CP_0430 | 89 | Hypothetical /ribosomal protein L28 |
| CPK_ORF00847 | 49 | CP_0420 | 49 | Hypothetical |
| CPK_ORF00857 | 59 | CP_0410 | 67 | Hypothetical |
| CPK_ORF00861 | 81 | CP_0406 | 81 | Hypothetical |
| CPK_ORF00865 | 52 |  |  | Hypothetical |
| CPK_ORF00866 | 53 | CP_0400 | 58 | Hypothetical |
| CPK_ORF00873 | 343 | CP_0392 | 344 | Hypothetical |
| CPK_ORF00875 | 51 |  |  | Hypothetical |
| CPK_ORF00879 | 117 | CP_0386 | 119 | Hypothetical |
| CPK_ORF00881 | 42 |  |  | Hypothetical |
| CPK_ORF00882 | 55 |  |  | Hypothetical |
| CPK_ORF00891 | 44 |  |  | Hypothetical |
| CPK_ORF00901 | 61 | CP_0364 | 61 | Hypothetical |
| CPK_ORF00931 | 39 | CP_0332 | 39 | Hypothetical |
| CPK_ORF00934 | 45 |  |  | Hypothetical |
| CPK_ORF00957 | 53 |  |  | Hypothetical |
| CPK_ORF00962 | 40 | CP_0304 | 40 | Hypothetical |
| CPK_ORF00964 | 52 |  |  | Hypothetical |
| CPK_ORF00969 | 690 | CP_0297 | 692 | Hypothetical |
| CPK_ORF00970 | 540* | CP_0295 | 629 | Hypothetical |
| CPK_ORF00971 | 1461* | CP_0295 | 629 | Hypothetical |
| CPK_ORF00972 | 40 |  |  | Hypothetical |
| CPK_ORF00973 | 684 | CP_0294 | 695 | Hypothetical |
| CPK_ORF00974 | 677 | CP_0291/292/293 | 265/283/129 | Hypothetical |
| CPK_ORF00975 | 45 |  |  | Hypothetical |
| CPK_ORF00977 | 2021* | CP_0290 | 672 | Hypothetical |
| CPK_ORF00979 | 675 | CP_0289/288 | 389/159 | Hypothetical |
| CPK_ORF00980 | 43 |  |  | Hypothetical |
| CPK_ORF00986 | 37 |  |  | Hypothetical |
| CPK_ORF00987 | 775 | CP_0282 | 775 | Hypothetical |
| CPK_ORF00997 | 536 |  |  | Hypothetical |
| CPK_ORF01009 | 41 | CP_0262 | 51 | Hypothetical |
| CPK_ORF01031 | 778 | CP_0237/236 | 520/279 | Hypothetical |
| CPK_ORF01039 | 37 |  |  | Hypothetical |
| CPK_ORF01079 | 42 |  |  | Hypothetical |
| CPK_ORF01081 | 49 | CP_0185 | 366 | Hypothetical |
| CPK_ORF01082 | 49 | CP_0184 | 49 | Hypothetical |
| CPK_ORF01102 | 655 | CP_0163 | 651 | Hypothetical |
| CPK_ORF01116 | 107 | CP_0148 | 107 | Hypothetical |
| No homology |  | CP_0597 | 91 | Hypothetical |

*An asterisk denotes the number of nucleotides – there is no amino acid translation
