# Supplementary figures and images for: Comparison of koala LPCoLN and human strains of Chlamydia pneumoniae highlights extended genetic diversity in the species
Source: BMC Genomics. 2010 Jul 21;11:442. doi: 10.1186/1471-2164-11-442 (PMC3091639; doi:10.1186/1471-2164-11-442)

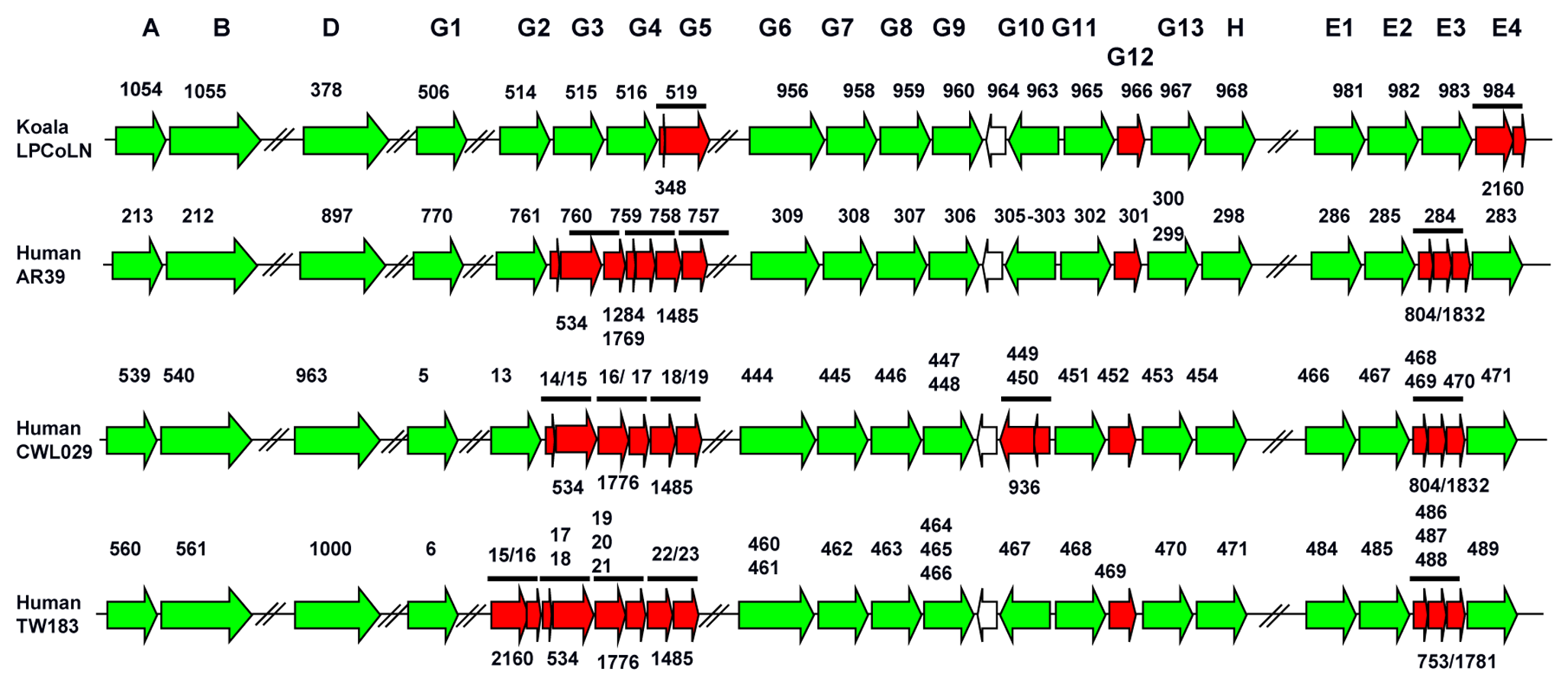

Supplement: Additional file 3 — Comparative analysis of the C. pneumoniae polymorphic membrane proteins (Pmps). A comparison of the 21 Pmps revealed a high degree of sequence polymorphism and indels between the koala LPCoLN and human AR39, CWL029 and TW183 isolates. The TW183 and J138 isolates were well-conserved and therefore, TW183 also represents J138 in this figure. Arrows indicate the direction of transcription: green arrows show typical pmp characteristics, red arrows represent pseudogenes (numbers below arrows indicate stop codon position), and white arrows represent proteins with no relation to pmps (note the same orientation as pmpG10). Dashed lines indicate truncated products. [file 1471-2164-11-442-S3.TIFF]

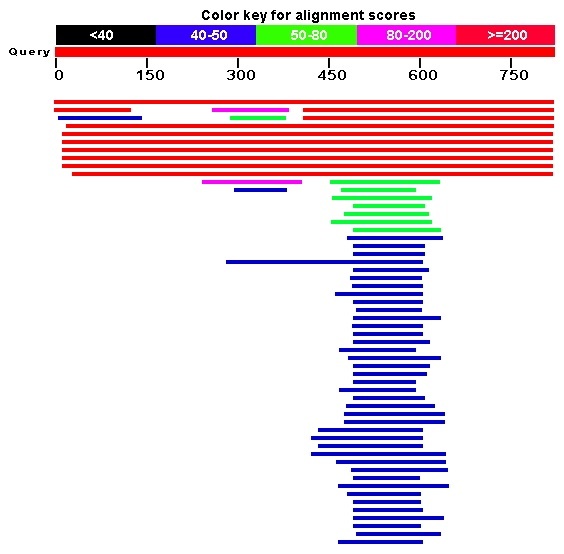

Supplement: Additional file 7 — Chlamydia MACPF. A BLAST alignment of the C. pneumoniae MACPF protein. From top to bottom: C. pneumoniae LPCoLN, C. pneumoniae J138, C. pneumoniae CWL029, C. felis FE/C-56, C. trachomatis A/HAR-13, C. trachomatis 6276, C. trachomatis D/UW-3/CX, C. trachomatis 70, C. trachomatis 434/Bu, C. muridarum Nigg, C. pneumoniae CWL029, C. abortus S26/3, C. felis Fe/C-56, Alcanivorax sp. DG881, Saccolglossus kowaleski, Theileria parva strain Muguga.... [file 1471-2164-11-442-S7.JPEG]

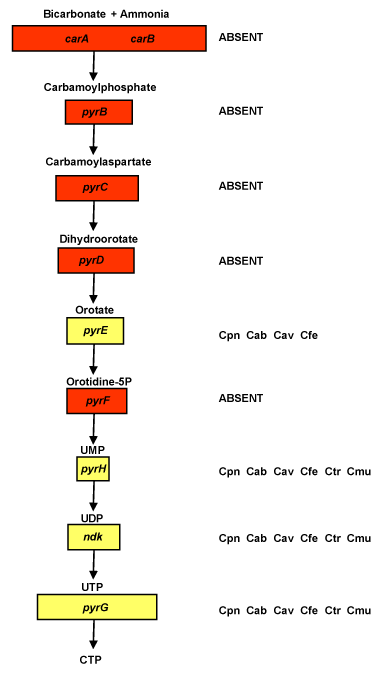

Supplement: Additional file 8 — Chlamydia has lost several steps in the pyrimidine biosynthesis pathway. All chlamydial genomes sequenced thus far, have lost the initial steps involved in pyrimidine biosynthesis. C. pneumoniae (Cpn), C. abortus (Cab), C. caviae (Cav) and C. felis (Cfe) contain a pyrE gene encoding an orotate phosphoribosyltransferase, while C. muridarum (Cmu) and C. trachomatis (Ctr) lack this gene. The next step in the pathway is via pyrF, which is absent from all chlamydial genomes. Interestingly, all six genomes have maintained the last three steps for the conversion of UMP into CTP. Adapted from Koonin and Galperin [66]. Gene names: carA, carbamoyl-phosphate synthase, small subunit; carB, carbamoyl-phosphate synthase, large subunit; pyrB, aspartate carbamoyltransferase; pyrC, dihydroorotase; pyrD, dihydroorotate dehydrogenase; pyrE, orotate phosphoribosyltransferase; pyrF, orotidine 5-phosphate decarboxylase; pyrH, uridylate kinase; ndk, nucleoside diphosphate kinase; pyrG, CTP synthase. Red boxes indicate gene loss, yellow boxes indicate the presence of a gene. [file 1471-2164-11-442-S8.TIFF]
