# Supplementary material for: Comparison of koala LPCoLN and human strains of Chlamydia pneumoniae highlights extended genetic diversity in the species
Source: BMC Genomics. 2010 Jul 21;11:442. doi: 10.1186/1471-2164-11-442 (PMC3091639; doi:10.1186/1471-2164-11-442)
Supplement: Additional file 6 — T3S ortholog comparisons. [file 1471-2164-11-442-S6.PDF]

LPCoLN genes predicted to be involved in T3S or homologous to T3S genes from other *Chlamydia* species:

ORF	Annotated
CPK_ORF00106	type II-III secretion system protein
CPK_ORF00107	serine-threonine-protein kinase
CPK_ORF00111	type III secretion apparatus H+-transporting two-sector ATPase
CPK_ORF00115	type III secretion apparatus protein, YscD-HrpQ family
CPK_ORF00116	type III secretion chaperone, CesT family
CPK_ORF00216	conserved hypothetical protein
CPK_ORF00217	conserved hypothetical protein
CPK_ORF00219	type III secretion low calcium response chaperone LcrH-SycD
CPK_ORF00231	bacterial export protein, fliR-mopE-spaR family
CPK_ORF00232	putative Yop translocation protein S
CPK_ORF00233	type III secretion apparatus protein, YscR-HrcR family
CPK_ORF00234	type III secretion apparatus protein, HrpE-YscL family
CPK_ORF00236	type III secretion apparatus lipoprotein, YscJ-HrcJ family
CPK_ORF00264	flagellum-specific ATP synthase FliI (fliI)
CPK_ORF00266	conserved hypothetical protein
CPK_ORF00430	Conserved hypothetical protein
CPK_ORF00445	conserved hypothetical protein
CPK_ORF00446	conserved hypothetical protein
CPK_ORF00447	putative type III secretion low calcium response chaperone LcrH-SycD
CPK_ORF00799	conserved hypothetical protein
CPK_ORF00800	conserved hypothetical protein
CPK_ORF00830	putative Yop translocation protein U
CPK_ORF00831	type III secretion protein AscV (ascV)
CPK_ORF00832	type III secretion regulator YopN-LcrE-InvE-MxiC
CPK_ORF00833	type III secretion chaperone, CesT family
CPK_ORF00897	type III secretion chaperone, CesT family

T3S apparatus genes:

% Identity
% Similarity

	1	2	3	4	5
1. ORF00106		99.5	99.5	99.5	99.5
2. CP_0044	99.5		100.0	100.0	100.0
3. CPn0702	99.5	100.0		100.0	100.0
4. CPj0702	99.5	100.0	100.0		100.0
5. CpB0729	99.5	100.0	100.0	100.0	

	1	2	3	4	5
1. ORF00111		99.8	99.8	99.8	99.8
2. CP_0039	99.8		100.0	100.0	100.0
3. CPn0707	99.8	100.0		100.0	100.0
4. CPj0707	99.8	100.0	100.0		100.0
5. CpB0734	99.8	100.0	100.0	100.0	

	1	2	3	4	5
1. ORF00115		99.6	99.6	99.6	99.0
2. CP_0034	99.6		100.0	100.0	99.4
3. CPn0712	99.6	100.0		100.0	99.4
4. CPj0712	99.6	100.0	100.0		99.4
5. CpB0739	99.0	99.4	99.4	99.4	

	1	2	3	4	5
1. ORF00231		99.4	99.4	99.4	99.4
2. CP_1048	99.4		100.0	100.0	100.0
3. CPn0823	99.4	100.0		100.0	100.0
4. CPj0823	99.4	100.0	100.0		100.0
5. CpB0852	99.4	100.0	100.0	100.0	

	1	2	3	4	5
1. ORF00232		100.0	100.0	100.0	100.0
2. CP_1047	100.0		100.0	100.0	100.0
3. CPn0824	100.0	100.0		100.0	100.0
4. CPj0824	100.0	100.0	100.0		100.0
5. CpB0853	100.0	100.0	100.0	100.0	

	1	2	3	4	5
1. ORF00233		99.2	99.2	99.2	99.2
2. CP_1046	99.2		100.0	100.0	100.0
3. CPn0825	99.2	100.0		100.0	100.0
4. CPj0825	99.2	100.0	100.0		100.0
5. CpB0854	99.2	100.0	100.0	100.0	

	1	2	3	4	5
1. ORF00234		99.6	99.6	99.6	99.6
2. CP_1045	99.6		100.0	100.0	100.0
3. CPn0826	99.6	100.0		100.0	100.0
4. CPj0826	99.6	100.0	100.0		100.0
5. CpB0855	99.6	100.0	100.0	100.0	

	1	2	3	4	5
1. ORF00236		98.2	98.2	98.2	98.2
2. CP_1043	98.2		100.0	100.0	100.0
3. CPn0828	98.2	100.0		100.0	100.0
4. CPj0828	98.2	100.0	100.0		100.0
5. CpB0857	98.2	100.0	100.0	100.0	

	1	2	3	4	5
1. ORF00830		99.8	99.7	99.8	99.8
2. CP_0435	99.8		99.9	100.0	100.0
3. CPn0322	99.7	99.9		99.9	99.9
4. CPj0322	99.8	100.0	99.9		100.0
5. CpB0332	99.8	100.0	99.9	100.0	

	1	2	3	4	5
1. ORF00831		99.7	99.7	99.7	99.7
2. CP_0434	99.7		100.0	100.0	100.0
3. CPn0323	99.7	100.0		100.0	100.0
4. CPj0323	99.7	100.0	100.0		100.0
5. CpB0333	99.7	100.0	100.0	100.0	

Flagellar homolog genes:

	1	2	3	4	5
1. ORF00264		99.8	99.9	99.8	99.9
2. CP_1011	99.8		99.9	99.9	99.9
3. CPn0858	99.9	99.9		99.8	100.0
4. CPj0858	99.8	99.9	99.8		99.8
5. CpB0887	99.9	99.9	100.0	99.8	

	1	2	3	4	5
1. ORF00266		99.7	99.7	99.7	99.7
2. CP_1009	99.7		100.0	100.0	100.0
3. CPn0860	99.7	100.0		100.0	100.0
4. CPj0860	99.7	100.0	100.0		100.0
5. CpB0889	99.7	100.0	100.0	100.0	

Chaperone genes:

	1	2	3	4	5
1. ORF00116		100.0	100.0	100.0	96.3
2. CP_0033	100.0		100.0	100.0	96.3
3. CPn0713	100.0	100.0		100.0	96.3
4. CPj0713	100.0	100.0	100.0		96.3
5. CpB0740	96.3	96.3	96.3	96.3	

	1	2	3	4	5
1. ORF00219		100.0	100.0	100.0	100.0
2. CP_1060	100.0		100.0	100.0	100.0
3. CPn0811	100.0	100.0		100.0	100.0
4. CPj0811	100.0	100.0	100.0		100.0
5. CpB0840	100.0	100.0	100.0	100.0	

	1	2	3	4	5
1. ORF00447		100.0	100.0	100.0	100.0
2. CP_0832	100.0		100.0	100.0	100.0
3. CPn1021	100.0	100.0		100.0	100.0
4. CPj1021	100.0	100.0	100.0		100.0
5. CpB1060	100.0	100.0	100.0	100.0	

	1	2	3	4	5
1. ORF00833		99.3	99.3	99.3	99.3
2. CP_0432	99.3		100.0	100.0	100.0
3. CPn0325	99.3	100.0		100.0	100.0
4. CPj0325	99.3	100.0	100.0		100.0
5. CpB0335	99.3	100.0	100.0	100.0	

	1	2	3	4	5
1. ORF00897		99.8	99.8	99.8	99.8
2. CP_0368	99.8		100.0	100.0	100.0
3. CPn0387	99.8	100.0		100.0	100.0
4. CPj0387	99.8	100.0	100.0		100.0
5. CpB0399	99.8	100.0	100.0	100.0	

Translocated protein genes:

	1	2	3	4	5
1. ORF00107		99.5	99.5	99.5	99.5
2. CP_0043	99.5		100.0	100.0	99.9
3. CPn0703	99.5	100.0		100.0	99.9
4. CPj0703	99.5	100.0	100.0		99.9
5. CpB0730	99.5	99.9	99.9	99.9	

	1	2	3	4	5
1. ORF00216		99.6	99.6	99.6	99.6
2. CP_1063	99.6		100.0	99.9	100.0
3. CPn0808	99.6	100.0		99.9	100.0
4. CPj0808	99.6	99.9	99.9		99.9
5. CpB0837	99.6	100.0	100.0	99.9	

	1	2	3	4	5
1. ORF00217		99.9	98.8	99.9	99.9
2. CP_1062	99.9		98.9	100.0	100.0
3. CPn0809	98.8	98.9		98.9	98.9
4. CPj0809	99.9	100.0	98.9		100.0
5. CpB0838	99.9	100.0	98.9	100.0	

	1	2	3	4	5
1. ORF00430		99.6	99.6	99.6	99.6
2. CP_0849	99.6		100.0	100.0	100.0
3. CPn1004	99.6	100.0		100.0	100.0
4. CPj1004	99.6	100.0	100.0		100.0
5. CpB1042	99.6	100.0	100.0	100.0	

	1	2	3	4	5
1. ORF00445		99.7	99.7	99.7	90.5
2. CP_0834	99.7		100.0	100.0	90.8
3. CPn1019	99.7	100.0		100.0	90.8
4. CPj1019	99.7	100.0	100.0		90.8
5. CpB1057	90.5	90.8	90.8	90.8	

	1	2	3	4	5
1. ORF00446		99.7	99.7	99.7	99.7
2. CP_0833	99.7		100.0	99.9	100.0
3. CPn1020	99.7	100.0		99.9	100.0
4. CPj1020	99.7	99.9	99.9		99.9
5. CpB1058	99.7	100.0	100.0	99.9	

	1	2	3	4	5
1. ORF00799		98.5	98.5	98.5	98.5
2. CP_0467	98.5		100.0	100.0	100.0
3. CPn0291	98.5	100.0		100.0	100.0
4. CPj0291	98.5	100.0	100.0		100.0
5. CpB0300	98.5	100.0	100.0	100.0	

	1	2	3	4	5
1. ORF00800		98.2	98.2	98.2	98.2
2. CP_0466	98.2		100.0	100.0	100.0
3. CPn0292	98.2	100.0		100.0	100.0
4. CPj0292	98.2	100.0	100.0		100.0
5. CpB0301	98.2	100.0	100.0	100.0	

	1	2	3	4	5
1. ORF00832		99.8	99.8	99.8	99.8
2. CP_0433	99.8		100.0	100.0	100.0
3. CPn0324	99.8	100.0		100.0	100.0
4. CPj0324	99.8	100.0	100.0		100.0
5. CpB0334	99.8	100.0	100.0	100.0	
