# Supplementary material for: Comparison of koala LPCoLN and human strains of Chlamydia pneumoniae highlights extended genetic diversity in the species
Source: BMC Genomics. 2010 Jul 21;11:442. doi: 10.1186/1471-2164-11-442 (PMC3091639; doi:10.1186/1471-2164-11-442)
Supplement: Additional file 9 — Sequence comparison of the chlamydial plasmid. Multiple sequence alignment of the predicted amino acid sequence from C. pneumoniae koala LPCoLN (pCpnKo), C. pneumoniae horse N16 (pCpnE1), C. psittaci avian N352 (pCpA1), C. felis feline Fe/C-56 (pCfe1), C. caviae guinea pig GPIC (pCpGP1), C. muridarum mouse Nigg (pMoPn) and C. trachomatis human serovars A (pCTA), B (pJALI), E (pSW2) and L1 (pLVG440). The sequences are well-conserved across species, indicating some degree of ancestry among them. The C. pneumoniae plasmid shared a close relationship with C. psittaci, C. caviae and C. felis, while C. muridarum and C. trachomatis were highly conserved. Predicted functions include plasmid replication (ORF1 and ORF2), double-stranded DNA unwinding (ORF3), chlamydial pathogenesis (ORF5) and regulation of partitioning and copy number (ORF7 and ORF8) [34,67]. The functions of ORF4 and ORF6 remain to be determined. [file 1471-2164-11-442-S9.PDF]

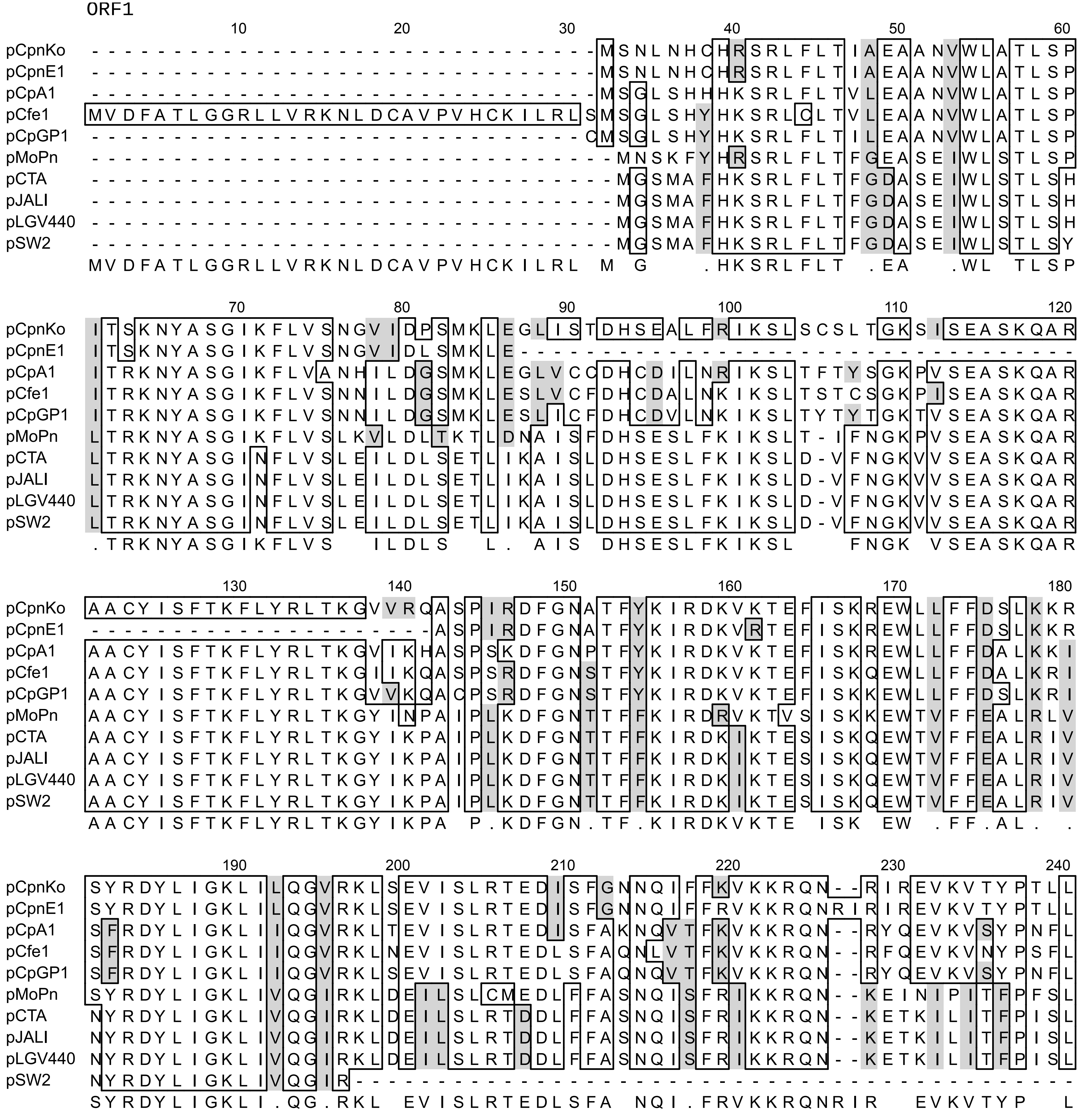
ORF1
pCpnKo
pCpnE1
pCpA1
pCfe1
pCpGP1
pMoPn
pCTA
pJALI
pLGV440
pSW2

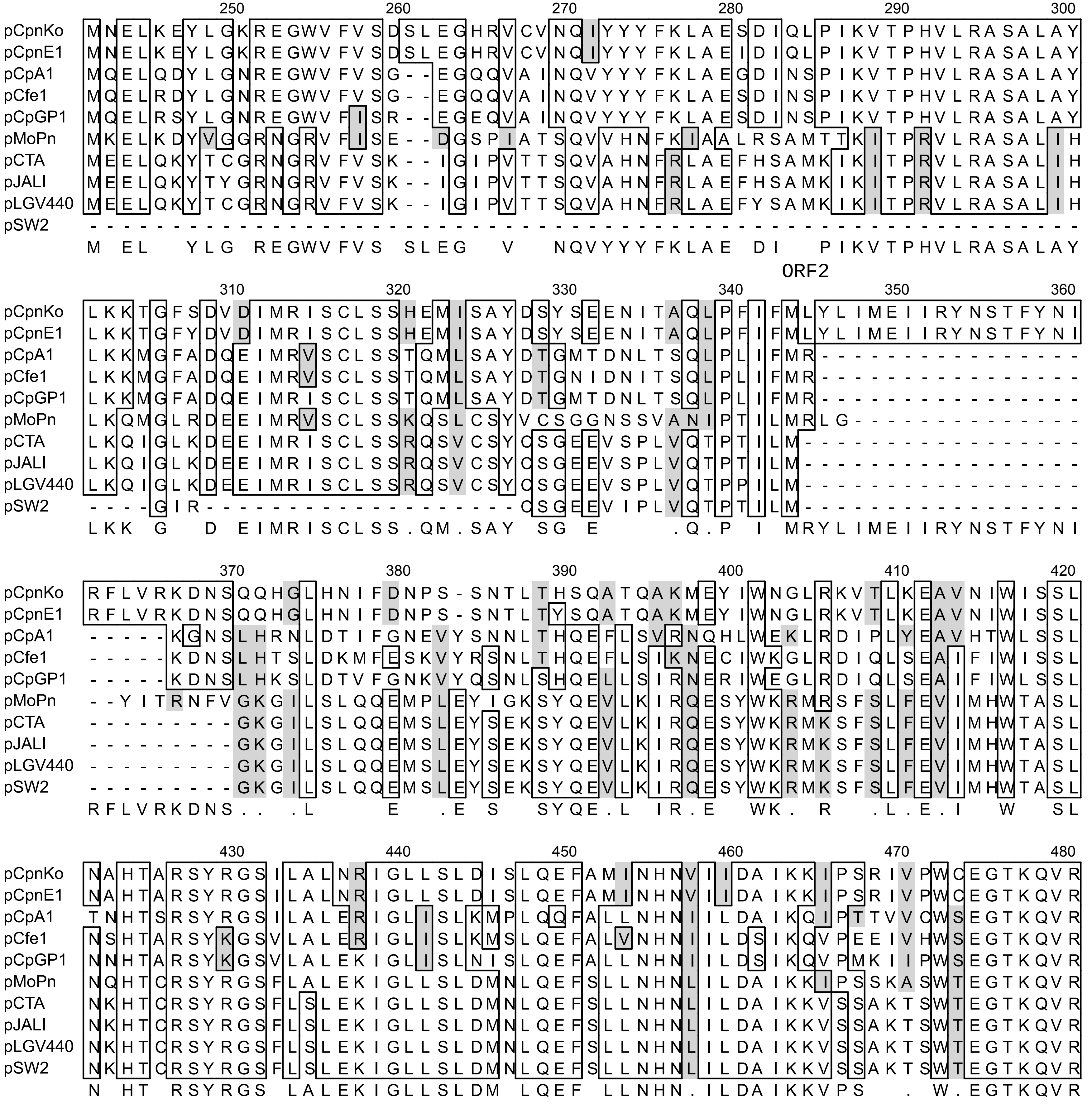
250 260 270 280 290 300
pCpnKo MNELKEYLGKREGWVFVSDSLEGHRVCVNQIYYYFKLAESDIQLPIKVTPHVLRASALAY
pCpnE1 MNELKEYLGKREGWVFVSDSLEGHRVCVNQIYYYFKLAEIDIQLPIKVTPHVLRASALAY
pCpA1 MQELQDYLGNREGWVFVSG--EGQQVAINQVYYYFKLAEGDINSPIKVTPHVLRASALAY
pCfe1 MQELRDYLGNREGWVFVSG--EGQQVAINQVYYYFKLAESDINSPIKVTPHVLRASALAY
pCpGP1 MQELRSYLGNREGWVFISR--EGEQVAINQVYYYFKLAESDINSPIKVTPHVLRASALAY
pMoPn MKELKDYVGGRNGRVFISE--DGSPIATSQVVHNFKIAALRSAMTTKITPRVLRASALIH
pCTA MEELQKYTCGRNGRVFVSK--IGIPVTTSQVAHNFRLAEFHSAMKIKITPRVLRASALIH
pJALI MEELQKYTYGRNGRVFVSK--IGIPVTTSQVAHNFRLAEFHSAMKIKITPRVLRASALIH
pLGV440 MEELQKYTCGRNGRVFVSK--IGIPVTTSQVAHNFRLAEFYSAMKIKITPRVLRASALIH
pSW2 --
M EL YLG REGWVFVS SLEG V NQVYYYFKLAE DI PIKVTPHVLRASALAY
ORF2
310 320 330 340 350 360
pCpnKo LKKTGFSDVDIMRISCLSSHEMISAYDSYSEENITAQLPFIFMLYLIMEIIRYNSTFYNI
pCpnE1 LKKTGFYDVDIMRISCLSSHEMISAYDSYSEENITAQLPFIFMLYLIMEIIRYNSTFYNI
pCpA1 LKKMGFADQEIMRVSCLSSTQMLSAYDTGMTDNLTSQLPLIFMR----------------
pCfe1 LKKMGFADQEIMRVSCLSSTQMLSAYDTGNIDNITSQLPLIFMR----------------
pCpGP1 LKKMGFADQEIMRISCLSSTQMLSAYDTGMTDNLTSQLPLIFMR----------------
pMoPn LKQMGLRDEEIMRVSCLSSKQSLCSYVCSGGNSSVANIPTILMRLG--------------
pCTA LKQIGLKDEEIMRISCLSSRQSVCSYCSGEEVSPLVQTPTILM-----------------
pJALI LKQIGLKDEEIMRISCLSSRQSVCSYCSGEEVSPLVQTPTILM-----------------
pLGV440 LKQIGLKDEEIMRISCLSSRQSVCSYCSGEEVSPLVQTPPILM-----------------
pSW2 ----GIR-------------------CSGEEVIPLVQTPTILM-----------------
LKK G D EIMRISCLSS.QM.SAY SG E .Q.P I MRYLIMEIIRYNSTFYNI
370 380 390 400 410 420
pCpnKo RFLVRKDNSQQHGLHNIFDNPS-SNTLTHSQATQAKMEYIWNGLRKVTLKEAVNIWISSL
pCpnE1 RFLVRKDNSQQHGLHNIFDNPS-SNTLTYSQATQAKMEYIWNGLRKVTLKEAVNIWISSL
pCpA1 -----KGNSLHRNLDTIFGNEVYSNNLTHQEFLSVRNQHLWEKLRDIPLYEAVHTWLSSL
pCfe1 -----KDNSLHTSLDKMFESKVYRSNLTHQEFLSIKNECIWKGLRDIQLSEAIFIWISSL
pCpGP1 -----KDNSLHKSLDTVFGNKVYQSNLSHQELLSIRNERIWEGLRDIQLSEAIFIWLSSL
pMoPn --YITRNFVGKGILSLQQEMPLEYIGKSYQEVLKIRQESYWKRMRSFSLFEVIMHWTASL
pCTA ---------GKGILSLQQEMSLEYSEKSYQEVLKIRQESYWKRMKSFSLFEVIMHWTASL
pJALI ---------GKGILSLQQEMSLEYSEKSYQEVLKIRQESYWKRMKSFSLFEVIMHWTASL
pLGV440 ---------GKGILSLQQEMSLEYSEKSYQEVLKIRQESYWKRMKSFSLFEVIMHWTASL
pSW2 ---------GKGILSLQQEMSLEYSEKSYQEVLKIRQESYWKRMKSFSLFEVIMHWTASL
RFLVRKDNS.. .L E .E S SYQE.L IR.E WK. R .L.E.I W SL
430 440 450 460 470 480
pCpnKo NAHTARSYRGSILALNRIGLLSLDISLQEFAMINHNVIIDAIKKIPSRIVPWCEGTKQVR
pCpnE1 NAHTARSYRGSILALNRIGLLSLDISLQEFAMINHNVIIDAIKKIPSRIVPWCEGTKQVR
pCpA1 TNHTSRSYRGSILALERIGLISLKMPLQQFALLNHNIILDAIKQIPTTVVCWSEGTKQVR
pCfe1 NSHTARSYKGSVLALERIGLISLKMSLQEFALVNHNIILDSIKQVPEEIVHWSEGTKQVR
pCpGP1 NNHTARSYKGSVLALEKIGLISLNISLQEFALLNHNIILDSIKQVPMKIIPWSEGTKQVR
pMoPn NQHTCRSYRGSFLALEKIGLLSLDMNLQEFSLLNHNLILDAIKKIPSSKASWTEGTKQVR
pCTA NKHTCRSYRGSFLSLEKIGLLSLDMNLQEFSLLNHNLILDAIKKVSSAKTSWTEGTKQVR
pJALI NKHTCRSYRGSFLSLEKIGLLSLDMNLQEFSLLNHNLILDAIKKVSSAKTSWTEGTKQVR
pLGV440 NKHTCRSYRGSFLSLEKIGLLSLDMNLQEFSLLNHNLILDAIKKVSSAKTSWTEGTKQVR
pSW2 NKHTCRSYRGSFLSLEKIGLLSLDMNLQEFSLLNHNLILDAIKKVSSAKTSWTEGTKQVR
N HT RSYRGS LALEKIGLLSLDM LQEF LLNHN.ILDAIKKVPS . W.EGTKQVR

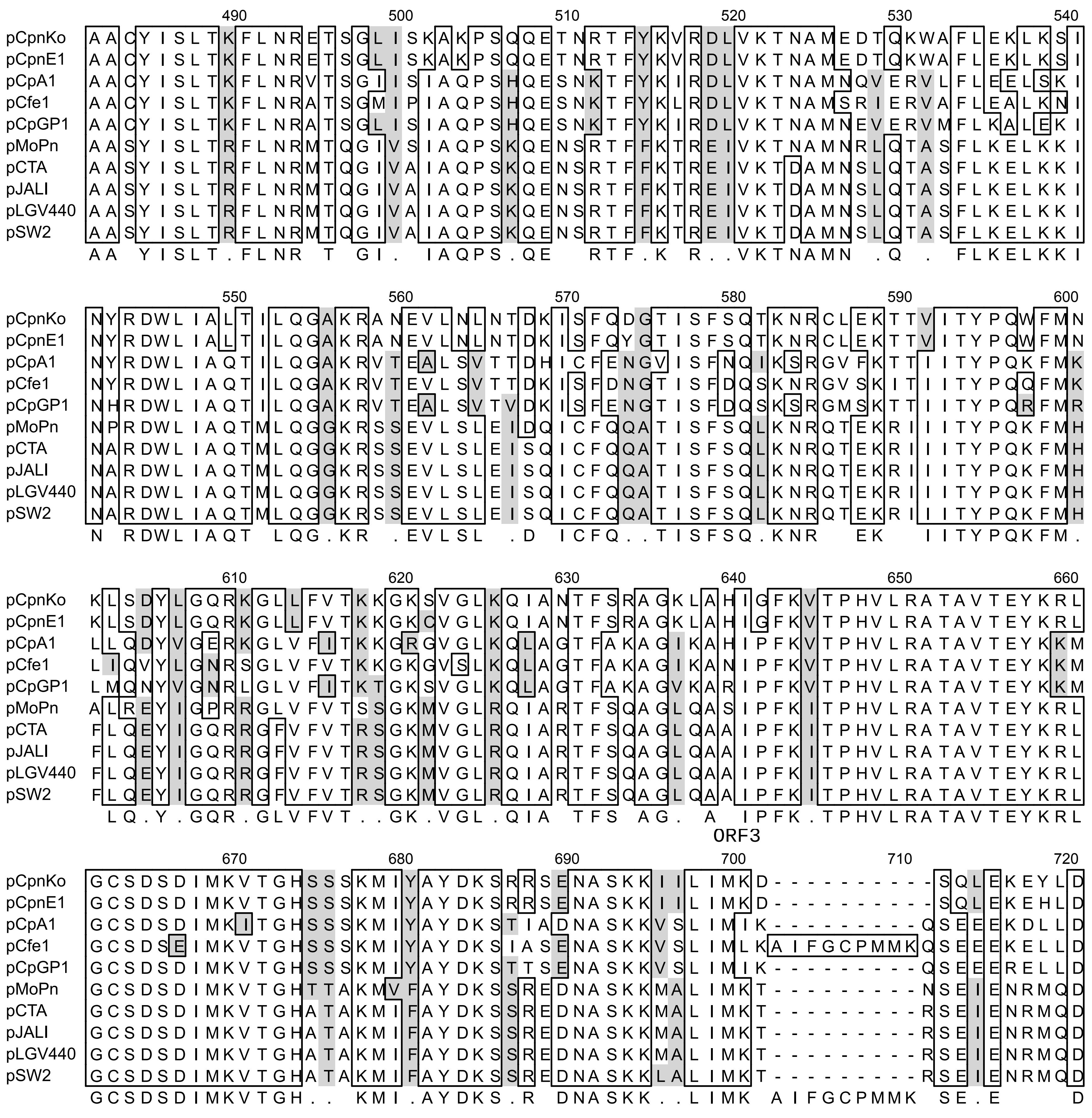
490 500 510 520 530 540
pCpnKo AACYISLTKFLNRETSGLISKAKPSQQETNRTFYKVRDLVKTNAMEDTQKWAFLEKLKSI
pCpnE1 AACYISLTKFLNRETSGLISKAKPSQQETNRTFYKVRDLVKTNAMEDTQKWAFLEKLKSI
pCpA1 AACYISLTKFLNRVTSGIISIAQPSHQESNKTFYKIRDLVKTNAMNQVERVLFLEELSKI
pCfe1 AACYISLTKFLNRATSGMIPIAQPSHQESNKTFYKLRDLVKTNAMSRIERVAFLEALKNI
pCpGP1 AACYISLTKFLNRATSGLISIAQPSHQESNKTFYKIRDLVKTNAMNEVERVMFLKALEKI
pMoPn AASYISLTRFLNRMTQGIVSIAQPSKQENSRTFFKTREIVKTNAMNRLQTASFLKELKKI
pCTA AASYISLTRFLNRMTQGIVAIAQPSKQENSRTFFKTREIVKTDAMNSLQTASFLKELKKI
pJALI AASYISLTRFLNRMTQGIVAIAQPSKQENSRTFFKTREIVKTDAMNSLQTASFLKELKKI
pLGV440 AASYISLTRFLNRMTQGIVAIAQPSKQENSRTFFKTREIVKTDAMNSLQTASFLKELKKI
pSW2 AASYISLTRFLNRMTQGIVAIAQPSKQENSRTFFKTREIVKTDAMNSLQTASFLKELKKI
AA YISLT.FLNR T GI. IAQPS.QE RTF.K R..VKTNAMN .Q . FLKELKKI
550 560 570 580 590 600
pCpnKo NYRDWLIALTILQGAKRANEVLNLNTDKISFQDGTISFSQTKNRCLEKTTVITYPQWFMN
pCpnE1 NYRDWLIALTILQGAKRANEVLNLNTDKISFQYGTISFSQTKNRCLEKTTVITYPQWFMN
pCpA1 NYRDWLIAQTILQGAKRVTEALSVTTDHICFENGVISFNQIKSRGVFKTTIITYPQKFMK
pCfe1 NYRDWLIAQTILQGAKRVTEVLSVTTDKISFDNGTISFDQSKNRGVSKITIITYPQQFMK
pCpGP1 NHRDWLIAQTILQGAKRVTEALSVTVDKISFENGTISFDQSKSRGMSKTTIITYPQRFMR
pMoPn NPRDWLIAQTMLQGGKRSSEVLSLEIDQICFQQATISFSQLKNRQTEKRIIITYPQKFMH
pCTA NARDWLIAQTMLQGGKRSSEVLSLEISQICFQQATISFSQLKNRQTEKRIIITYPQKFMH
pJALI NARDWLIAQTMLQGGKRSSEVLSLEISQICFQQATISFSQLKNRQTEKRIIITYPQKFMH
pLGV440 NARDWLIAQTMLQGGKRSSEVLSLEISQICFQQATISFSQLKNRQTEKRIIITYPQKFMH
pSW2 NARDWLIAQTMLQGGKRSSEVLSLEISQICFQQATISFSQLKNRQTEKRIIITYPQKFMH
N RDWLIAQT LQG.KR .EVLSL .D ICFQ..TISFSQ.KNR EK IITYPQKFM.
610 620 630 640 650 660
pCpnKo KLSDYLGQRKGLLFVTKKGKSVGLKQIANTFSRAGKLAHIGFKVTPHVLRATAVTEYKRL
pCpnE1 KLSDYLGQRKGLLFVTKKGKCVGLKQIANTFSRAGKLAHIGFKVTPHVLRATAVTEYKRL
pCpA1 LLQDYVGERKGLVFITKKGRGVGLKQLAGTFAKAGIKAHIPFKVTPHVLRATAVTEYKKM
pCfe1 LIQVYLGNRSGLVFVTKKGKGVSLKQLAGTFAKAGIKANIPFKVTPHVLRATAVTEYKKM
pCpGP1 LMQNYVGNRLGLVFITKTGKSVGLKQLAGTFAKAGVKARIPFKVTPHVLRATAVTEYKKM
pMoPn ALREYIGPRRGLVFVTSSGKMVGLRQIARTFSQAGLQASIPFKITPHVLRATAVTEYKRL
pCTA FLQEYIGQRRGFVFVTRSGKMVGLRQIARTFSQAGLQAAIPFKITPHVLRATAVTEYKRL
pJALI FLQEYIGQRRGFVFVTRSGKMVGLRQIARTFSQAGLQAAIPFKITPHVLRATAVTEYKRL
pLGV440 FLQEYIGQRRGFVFVTRSGKMVGLRQIARTFSQAGLQAAIPFKITPHVLRATAVTEYKRL
pSW2 FLQEYIGQRRGFVFVTRSGKMVGLRQIARTFSQAGLQAAIPFKITPHVLRATAVTEYKRL
LQ.Y.GQR.GLVFVT..GK.VGL.QIA TFS AG. A IPFK.TPHVLRATAVTEYKRL
ORF3
670 680 690 700 710 720
pCpnKo GCSDSDIMKVTGHSSSKMIYAYDKSRRSENASKKIILIMKD----------SQLEKEYLD
pCpnE1 GCSDSDIMKVTGHSSSKMIYAYDKSRRSENASKKIILIMKD----------SQLEKEHLD
pCpA1 GCSDSDIMKITGHSSSKMIYAYDKSTIADNASKKVSLIMIK---------QSEEEKDLLD
pCfe1 GCSDSEIMKVTGHSSSKMIYAYDKSIASENASKKVSLIMLKAIFGCPMMKQSEEEKELLD
pCpGP1 GCSDSDIMKVTGHSSSKMIYAYDKSTTSENASKKVSLIMIK---------QSEEERELLD
pMoPn GCSDSDIMKVTGHTTAKMVFAYDKSSREDNASKKMALIMKT---------NSEIENRMQD
pCTA GCSDSDIMKVTGHATAKMIFAYDKSSREDNASKKMALIMKT---------RSEIENRMQD
pJALI GCSDSDIMKVTGHATAKMIFAYDKSSREDNASKKMALIMKT---------RSEIENRMQD
pLGV440 GCSDSDIMKVTGHATAKMIFAYDKSSREDNASKKMALIMKT---------RSEIENRMQD
pSW2 GCSDSDIMKVTGHATAKMIFAYDKSSREDNASKKLALIMKT---------RSEIENRMQD
GCSDSDIMKVTGH.. KMI.AYDKS.R DNASKK..LIMK AIFGCPMMK SE.E D

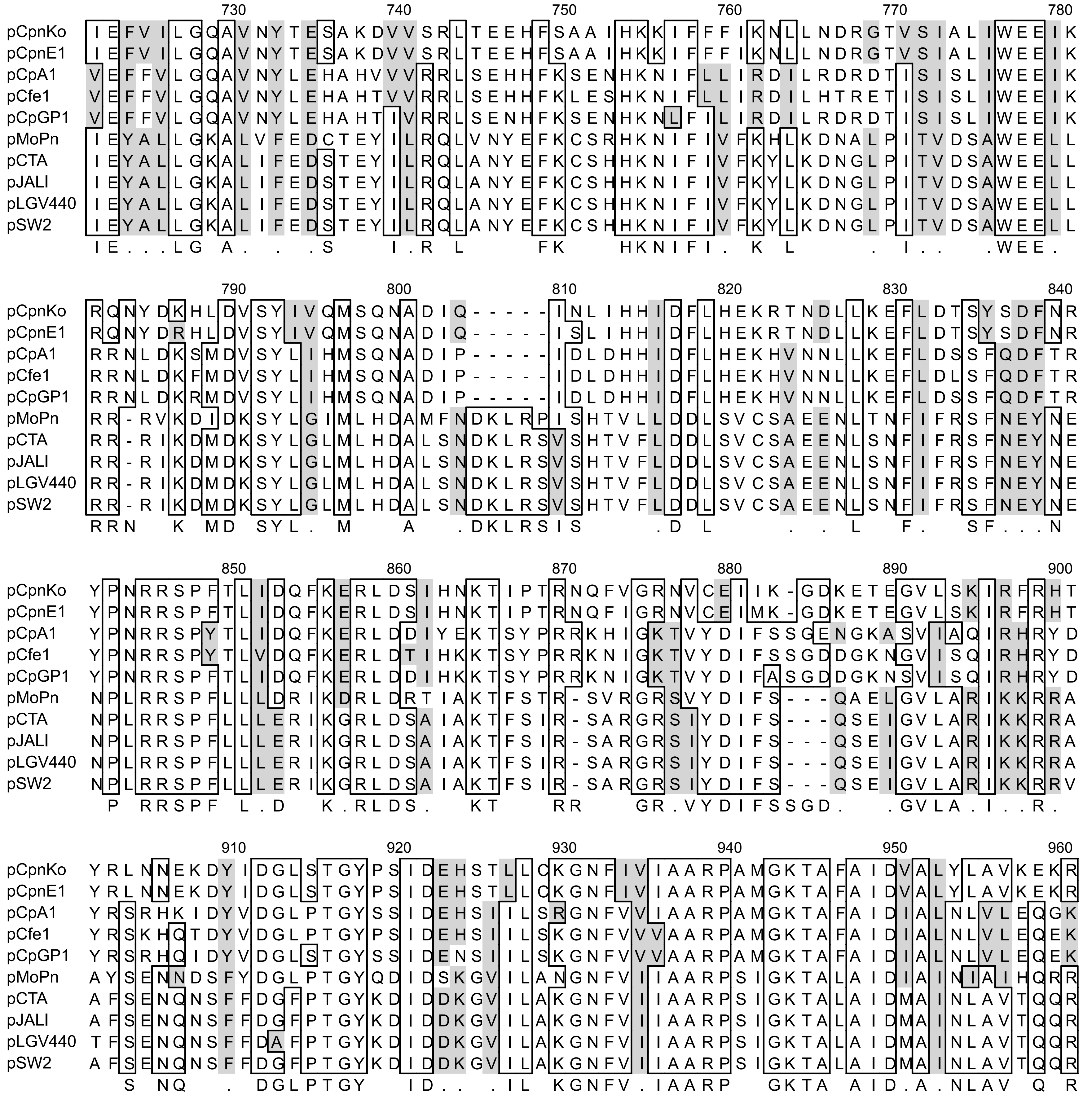
730 740 750 760 770 780
pCpnKo IEFVILGQAVNYTESAKDVVSRLTEEHFSAAIHKKIFFFIKNLLNDRGTVSIALIWEEIK
pCpnE1 IEFVILGQAVNYTESAKDVVSRLTEEHFSAAIHKKIFFFIKNLLNDRGTVSIALIWEEIK
pCpA1 VEFFVLGQAVNYLEHAHVVVRRLSEHHFKSENHKNIFLLIRDILRDRDTISISLIWEEIK
pCfe1 VEFFVLGQAVNYLEHAHTVVRRLSEHHFKLESHKNIFLLIRDILHTRETISISLIWEEIK
pCpGP1 VEFFVLGQAVNYLEHAHTIVRRLSENHFKSENHKNLFILIRDILRDRDTISISLIWEEIK
pMoPn IEYALLGKALVFEDCTEYILRQLVNYEFKCSRHKNIFIVFKHLKDNALPITVDSAWEELL
pCTA IEYALLGKALIFEDSTEYILRQLANYEFKCSHHKNIFIVFKYLKDNGLPITVDSAWEELL
pJALI IEYALLGKALIFEDSTEYILRQLANYEFKCSHHKNIFIVFKYLKDNGLPITVDSAWEELL
pLGV440 IEYALLGKALIFEDSTEYILRQLANYEFKCSHHKNIFIVFKYLKDNGLPITVDSAWEELL
pSW2 IEYALLGKALIFEDSTEYILRQLANYEFKCSHHKNIFIVFKYLKDNGLPITVDSAWEELL
IE...LG A. . .S I.R L FK HKNIFI. K L . I.. .WEE.
790 800 810 820 830 840
pCpnKo RQNYDKHLDVSYIVQMSQNADIQ-----INLIHHIDFLHEKRTNDLLKEFLDTSYSDFNR
pCpnE1 RQNYDRHLDVSYIVQMSQNADIQ-----ISLIHHIDFLHEKRTNDLLKEFLDTSYSDFNR
pCpA1 RRNLDKSMDVSYLIHMSQNADIP-----IDLDHHIDFLHEKHVNNLLKEFLDSSFQDFTR
pCfe1 RRNLDKFMDVSYLIHMSQNADIP-----IDLDHHIDFLHEKHVNNLLKEFLDLSFQDFTR
pCpGP1 RRNLDKRMDVSYLIHMSQNADIP-----IDLDHHIDFLHEKHVNNLLKEFLDSSFQDFTR
pMoPn RR-RVKDIDKSYLGIMLHDAMFNDKLRPISHTVLLDDLSVCSAEENLTNFIFRSFNEYNE
pCTA RR-RIKDMDKSYLGLMLHDALSNDKLRSVSHTVFLDDLSVCSAEENLSNFIFRSFNEYNE
pJALI RR-RIKDMDKSYLGLMLHDALSNDKLRSVSHTVFLDDLSVCSAEENLSNFIFRSFNEYNE
pLGV440 RR-RIKDMDKSYLGLMLHDALSNDKLRSVSHTVFLDDLSVCSAEENLSNFIFRSFNEYNE
pSW2 RR-RIKDMDKSYLGLMLHDALSNDKLRSVSHTVFLDDLSVCSAEENLSNFIFRSFNEYNE
RRN K MD SYL. M A .DKLRSIS .D L . . L F. SF...N
850 860 870 880 890 900
pCpnKo YPNRRSPFTLIDQFKERLDSIHNKTIPTRNQFVGRNVCEIIK-GDKETEGVLSKIRFRHT
pCpnE1 YPNRRSPFTLIDQFKERLDSIHNKTIPTRNQFIGRNVCEIMK-GDKETEGVLSKIRFRHT
pCpA1 YPNRRSPYTLIDQFKERLDDIYEKTSYPRRKHIGKTVYDIFSSGENGKASVIAQIRHRYD
pCfe1 YPNRRSPYTLVDQFKERLDTIHKKTSYPRRKNIGKTVYDIFSSGDDGKNGVISQIRHRYD
pCpGP1 YPNRRSPFTLIDQFKERLDDIHKKTSYPRRKNIGKTVYDIFASGDDGKNSVISQIRHRYD
pMoPn NPLRRSPFLLLDRIKDRLDRTIAKTFSTR-SVRGRSVYDIFS---QAELGVLARIKKRRA
pCTA NPLRRSPFLLLERIKGRLDSAIAKTFSIR-SARGRSIYDIFS---QSEIGVLARIKKRRA
pJALI NPLRRSPFLLLERIKGRLDSAIAKTFSIR-SARGRSIYDIFS---QSEIGVLARIKKRRA
pLGV440 NPLRRSPFLLLERIKGRLDSAIAKTFSIR-SARGRSIYDIFS---QSEIGVLARIKKRRA
pSW2 NPLRRSPFLLLERIKGRLDSAIAKTFSIR-SARGRSIYDIFS---QSEIGVLARIKKRRV
P RRSPF L.D K.RLDS. KT RR GR.VYDIFSSGD. .GVLA.I..R.
910 920 930 940 950 960
pCpnKo YRLNNEKDYIDGLSTGYPSIDEHSTLLCKGNFIVIAARPAMGKTAFAIDVALYLAVKEKR
pCpnE1 YRLNNEKDYIDGLSTGYPSIDEHSTLLCKGNFIVIAARPAMGKTAFAIDVALYLAVKEKR
pCpA1 YRSRHKIDYVDGLPTGYSSIDEHSIILSRGNFVVIAARPAMGKTAFAIDIALNLVLEQGK
pCfe1 YRSKHQTDYVDGLPTGYPSIDEHSIILSKGNFVVVAARPAMGKTAFAIDIALNLVLEQEK
pCpGP1 YRSRHQIDYVDGLSTGYSSIDENSIILSKGNFVVVAARPAMGKTAFAIDIALNLVLEQEK
pMoPn AYSENNDSFYDGLPTGYQDIDSKGVILANGNFVIIAARPSIGKTALAIDIAINIAIHQRR
pCTA AFSENQNSFFDGFPTGYKDIDDKGVILAKGNFVIIAARPSIGKTALAIDMAINLAVTQQR
pJALI AFSENQNSFFDGFPTGYKDIDDKGVILAKGNFVIIAARPSIGKTALAIDMAINLAVTQQR
pLGV440 TFSENQNSFFDAFPTGYKDIDDKGVILAKGNFVIIAARPSIGKTALAIDMAINLAVTQQR
pSW2 AFSENQNSFFDGFPTGYKDIDDKGVILAKGNFVIIAARPSIGKTALAIDMAINLAVTQQR
S NQ . DGLPTGY ID.. .IL KGNFV.IAARP GKTA AID.A.NLAV Q R

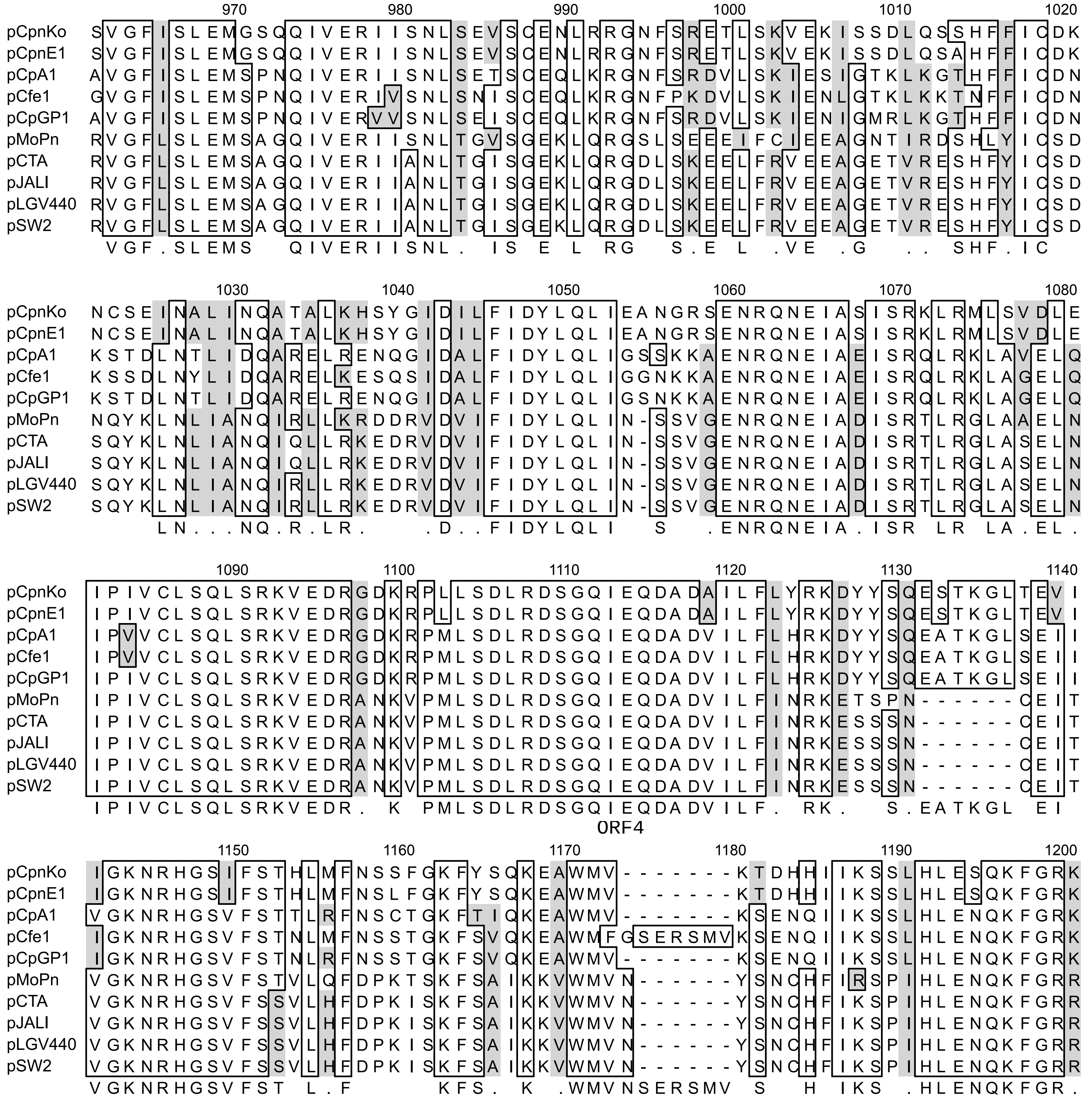
970 980 990 1000 1010 1020
pCpnKo SVGFISLEMGSQQIVERIISNLSEVSCENLRRGNFSRETLSKVEKISSDLQSSHFFICDK
pCpnE1 SVGFISLEMGSQQIVERIISNLSEVSCENLRRGNFSRETLSKVEKISSDLQSAHFFICDK
pCpA1 AVGFISLEMSPNQIVERIISNLSETSCEQLKRGNFSRDVLSKIESIGTKLKGTHFFICDN
pCfe1 GVGFISLEMSPNQIVERIVSNLSNISCEQLKRGNFPKDVLSKIENLGTKLKKTNFFICDN
pCpGP1 AVGFISLEMSPNQIVERVVSNLSEISCEQLKRGNFSRDVLSKIENIGMRLKGTHFFICDN
pMoPn RVGFLSLEMSAGQIVERIISNLTGVSGEKLQRGSLSEEEIFCIEEAGNTIRDSHLYICSD
pCTA RVGFLSLEMSAGQIVERIIANLTGISGEKLQRGDLSKEELFRVEEAGETVRESHFYICSD
pJALI RVGFLSLEMSAGQIVERIIANLTGISGEKLQRGDLSKEELFRVEEAGETVRESHFYICSD
pLGV440 RVGFLSLEMSAGQIVERIIANLTGISGEKLQRGDLSKEELFRVEEAGETVRESHFYICSD
pSW2 RVGFLSLEMSAGQIVERIIANLTGISGEKLQRGDLSKEELFRVEEAGETVRESHFYICSD
VGF.SLEMS QIVERIISNL. IS E L RG S.E L .VE .G .. SHF.IC
1030 1040 1050 1060 1070 1080
pCpnKo NCSEINALINQATALKHSYGIDILFIDYLQLIEANGRSENRQNEIASISRKLRMLSVDLE
pCpnE1 NCSEINALINQATALKHSYGIDILFIDYLQLIEANGRSENRQNEIASISRKLRMLSVDLE
pCpA1 KSTDLNTLIDQARELRENQGIDALFIDYLQLIGSSKKAENRQNEIAEISRQLRKLAVELQ
pCfe1 KSSDLNYLIDQARELKESQSIDALFIDYLQLIGGNKKAENRQNEIAEISRQLRKLAGELQ
pCpGP1 KSTDLNTLIDQARELRENQGIDALFIDYLQLIGSNKKAENRQNEIAEISRQLRKLAGELQ
pMoPn NQYKLNLIANQIRLLKRDDRVDVIFIDYLQLIN-SSVGENRQNEIADISRTLRGLAAELN
pCTA SQYKLNLIANQIQLLRKEDRVDVIFIDYLQLIN-SSVGENRQNEIADISRTLRGLASELN
pJALI SQYKLNLIANQIQLLRKEDRVDVIFIDYLQLIN-SSVGENRQNEIADISRTLRGLASELN
pLGV440 SQYKLNLIANQIRLLRKEDRVDVIFIDYLQLIN-SSVGENRQNEIADISRTLRGLASELN
pSW2 SQYKLNLIANQIRLLRKEDRVDVIFIDYLQLIN-SSVGENRQNEIADISRTLRGLASELN
LN...NQ.R.LR. .D..FIDYLQLI S .ENRQNEIA.ISR LR LA.EL.
1090 1100 1110 1120 1130 1140
pCpnKo IPIVCLSQLSRKVEDRGDKRPLLSDLRDSGQIEQDADAILFLYRKDYYSQESTKGLTEVI
pCpnE1 IPIVCLSQLSRKVEDRGDKRPLLSDLRDSGQIEQDADAILFLYRKDYYSQESTKGLTEVI
pCpA1 IPVVCLSQLSRKVEDRGDKRPMLSDLRDSGQIEQDADVILFLHRKDYYSQEATKGLSEII
pCfe1 IPVVCLSQLSRKVEDRGDKRPMLSDLRDSGQIEQDADVILFLHRKDYYSQEATKGLSEII
pCpGP1 IPIVCLSQLSRKVEDRGDKRPMLSDLRDSGQIEQDADVILFLHRKDYYSQEATKGLSEII
pMoPn IPIVCLSQLSRKVEDRANKVPMLSDLRDSGQIEQDADVILFINRKETSPN------CEIT
pCTA IPIVCLSQLSRKVEDRANKVPMLSDLRDSGQIEQDADVILFINRKESSSN------CEIT
pJALI IPIVCLSQLSRKVEDRANKVPMLSDLRDSGQIEQDADVILFINRKESSSN------CEIT
pLGV440 IPIVCLSQLSRKVEDRANKVPMLSDLRDSGQIEQDADVILFINRKESSSN------CEIT
pSW2 IPIVCLSQLSRKVEDRANKVPMLSDLRDSGQIEQDADVILFINRKESSSN------CEIT
IPIVCLSQLSRKVEDR. K PMLSDLRDSGQIEQDADVILF. RK. S.EATKGL EI
ORF4
1150 1160 1170 1180 1190 1200
pCpnKo IGKNRHGSIFSTHLMFNSSFGKFYSQKEAWMV--------KTDHHIIKSSLHLESQKFGRK
pCpnE1 IGKNRHGSIFSTHLMFNSLFGKFYSQKEAWMV--------KTDHHIIKSSLHLESQKFGRK
pCpA1 VGKNRHGSVFSTTLRFNSCTGKFTIQKEAWMV--------KSENQIIKSSLHLENQKFGRK
pCfe1 IGKNRHGSVFSTNLMFNSSTGKFSVQKEAWMFGSERSMVKSENQIIKSSLHLENQKFGRK
pCpGP1 IGKNRHGSVFSTNLRFNSSTGKFSVQKEAWMV--------KSENQIIKSSLHLENQKFGRK
pMoPn VGKNRHGSVFSTVLQFDPKTSKFSAIKKVWMVN-------YSNCHFIRSPIHLENQKFGRR
pCTA VGKNRHGSVFSSVLHFDPKISKFSAIKKVWMVN-------YSNCHFIKSPIHLENQKFGRR
pJALI VGKNRHGSVFSSVLHFDPKISKFSAIKKVWMVN-------YSNCHFIKSPIHLENQKFGRR
pLGV440 VGKNRHGSVFSSVLHFDPKISKFSAIKKVWMVN-------YSNCHFIKSPIHLENQKFGRR
pSW2 VGKNRHGSVFSSVLHFDPKISKFSAIKKVWMVN-------YSNCHFIKSPIHLENQKFGRR
VGKNRHGSVFST L.F KFS. K .WMVNSERSMV S H IKS .HLENQKFGR.

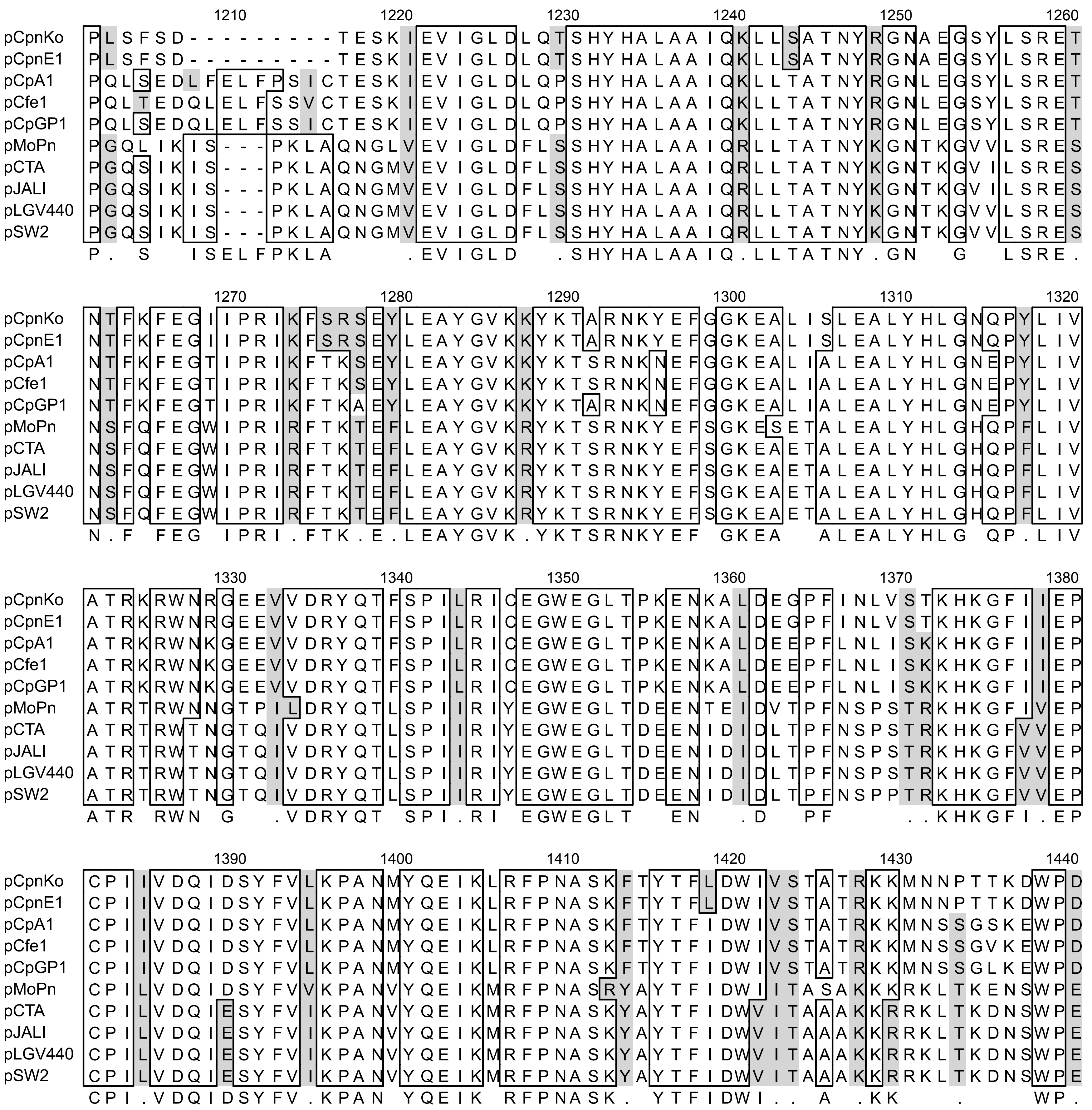
1210 1220 1230 1240 1250 1260
pCpnKo PLSFSD----------TESKIEVIGLDLQTSHYHALAAIQKLLSATNYRGNAEGSYLSRET
pCpnE1 PLSFSD----------TESKIEVIGLDLQTSHYHALAAIQKLLSATNYRGNAEGSYLSRET
pCpA1 PQLSEDLFELFPSICTESKIEVIGLDLQPSHYHALAAIQKLLTATNYRGNLEGSYLSRET
pCfe1 PQLTEDQLELFSSVCTESKIEVIGLDLQPSHYHALAAIQKLLTATNYRGNLEGSYLSRET
pCpGP1 PQLSEDQLELFSSICTESKIEVIGLDLQPSHYHALAAIQKLLTATNYRGNLEGSYLSRET
pMoPn PGQLIKIS---PKLAQNGLVEVIGLDFLSSHYHALAAIQRLLTATNYKGNTKGVVLSRES
pCTA PGQSIKIS---PKLAQNGMVEVIGLDFLSSHYHALAAIQRLLTATNYKGNTKGVILSRES
pJALI PGQSIKIS---PKLAQNGMVEVIGLDFLSSHYHALAAIQRLLTATNYKGNTKGVILSRES
pLGV440 PGQSIKIS---PKLAQNGMVEVIGLDFLSSHYHALAAIQRLLTATNYKGNTKGVVLSRES
pSW2 PGQSIKIS---PKLAQNGMVEVIGLDFLSSHYHALAAIQRLLTATNYKGNTKGVVLSRES
P . S ISELFPKLA .EVIGLD .SHYHALAAIQ.LLTATNY.GN G LSRE.
1270 1280 1290 1300 1310 1320
pCpnKo NTFKFEGIIPRIKFSRSEYLEAYGVKKYKTARNKYEFGGKEALISLEALYHLGNQPYLIV
pCpnE1 NTFKFEGIIPRIKFSRSEYLEAYGVKKYKTARNKYEFGGKEALISLEALYHLGNQPYLIV
pCpA1 NTFKFEGTIPRIKFTKSEYLEAYGVKKYKTSRNKNEFGGKEALIALEALYHLGNEPYLIV
pCfe1 NTFKFEGTIPRIKFTKSEYLEAYGVKKYKTSRNKNEFGGKEALIALEALYHLGNEPYLIV
pCpGP1 NTFKFEGTIPRIKFTKAEYLEAYGVKKYKTARNKNEFGGKEALIALEALYHLGNEPYLIV
pMoPn NSFQFEGWIPRIRFTKTEFLEAYGVKRYKTSRNKYEFSGKESETALEALYHLGHQPFLIV
pCTA NSFQFEGWIPRIRFTKTEFLEAYGVKRYKTSRNKYEFSGKEAETALEALYHLGHQPFLIV
pJALI NSFQFEGWIPRIRFTKTEFLEAYGVKRYKTSRNKYEFSGKEAETALEALYHLGHQPFLIV
pLGV440 NSFQFEGWIPRIRFTKTEFLEAYGVKRYKTSRNKYEFSGKEAETALEALYHLGHQPFLIV
pSW2 NSFQFEGWIPRIRFTKTEFLEAYGVKRYKTSRNKYEFSGKEAETALEALYHLGHQPFLIV
N.F FEG IPRI.FTK.E.LEAYGVK.YKTSRNKYEF GKEA ALEALYHLG QP.LIV
1330 1340 1350 1360 1370 1380
pCpnKo ATRKRWNRGEEVVDRYQTFSPILRICEGWEGLTPKENKALDEGPFINLVSTKHKGFIIEP
pCpnE1 ATRKRWNRGEEVVDRYQTFSPILRICEGWEGLTPKENKALDEGPFINLVSTKHKGFIIEP
pCpA1 ATRKRWNKGEEVVDRYQTFSPILRICEGWEGLTPKENKALDEEPFLNLISKKHKGFIIEP
pCfe1 ATRKRWNKGEEVVDRYQTFSPILRICEGWEGLTPKENKALDEEPFLNLISKKHKGFIIEP
pCpGP1 ATRKRWNKGEEVVDRYQTFSPILRICEGWEGLTPKENKALDEEPFLNLISKKHKGFIIEP
pMoPn ATRTRWNNGTPILDRYQTLSPIIRIYEGWEGLTDEENTEIDVTPFNSPSTRKHKGFIVEP
pCTA ATRTRWTNGTQIVDRYQTLSPIIRIYEGWEGLTDEENIDIDLTPFNSPSTRKHKGFVVEP
pJALI ATRTRWTNGTQIVDRYQTLSPIIRIYEGWEGLTDEENIDIDLTPFNSPSTRKHKGFVVEP
pLGV440 ATRTRWTNGTQIVDRYQTLSPIIRIYEGWEGLTDEENIDIDLTPFNSPSTRKHKGFVVEP
pSW2 ATRTRWTNGTQIVDRYQTLSPIIRIYEGWEGLTDEENIDIDLTPFNSPPTRKHKGFVVEP
ATR RWN G .VDRYQT SPI.RI EGWEGLT EN .D PF ..KHKGFI.EP
1390 1400 1410 1420 1430 1440
pCpnKo CPIIVDQIDSYFVLKPANMYQEIKLRFPNASKFTYTFLDWIVSTATRKKMNNPTTKDWPD
pCpnE1 CPIIVDQIDSYFVLKPANMYQEIKLRFPNASKFTYTFLDWIVSTATRKKMNNPTTKDWPD
pCpA1 CPIIVDQIDSYFVLKPANMYQEIKLRFPNASKFTYTFIDWIVSTATRKKMNSSGSKEWPD
pCfe1 CPIIVDQIDSYFVLKPANMYQEIKLRFPNASKFTYTFIDWIVSTATRKKMNSSGVKEWPD
pCpGP1 CPIIVDQIDSYFVLKPANMYQEIKLRFPNASKFTYTFIDWIVSTATRKKMNSSGLKEWPD
pMoPn CPILVDQIDSYFVVKPANVYQEIKMRFPNASRYAYTFIDWIITASAKKKRKLTKENSWPE
pCTA CPILVDQIESYFVIKPANVYQEIKMRFPNASKYAYTFIDWVITAAAKKRRKLTKDNSWPE
pJALI CPILVDQIESYFVIKPANVYQEIKMRFPNASKYAYTFIDWVITAAAKKRRKLTKDNSWPE
pLGV440 CPILVDQIESYFVIKPANVYQEIKMRFPNASKYAYTFIDWVITAAAKKRRKLTKDNSWPE
pSW2 CPILVDQIESYFVIKPANVYQEIKMRFPNASKYAYTFIDWVITAAAKKRRKLTKDNSWPE
CPI.VDQIDSYFV.KPAN YQEIK RFPNASK. YTFIDWI.. A .KK . WP.

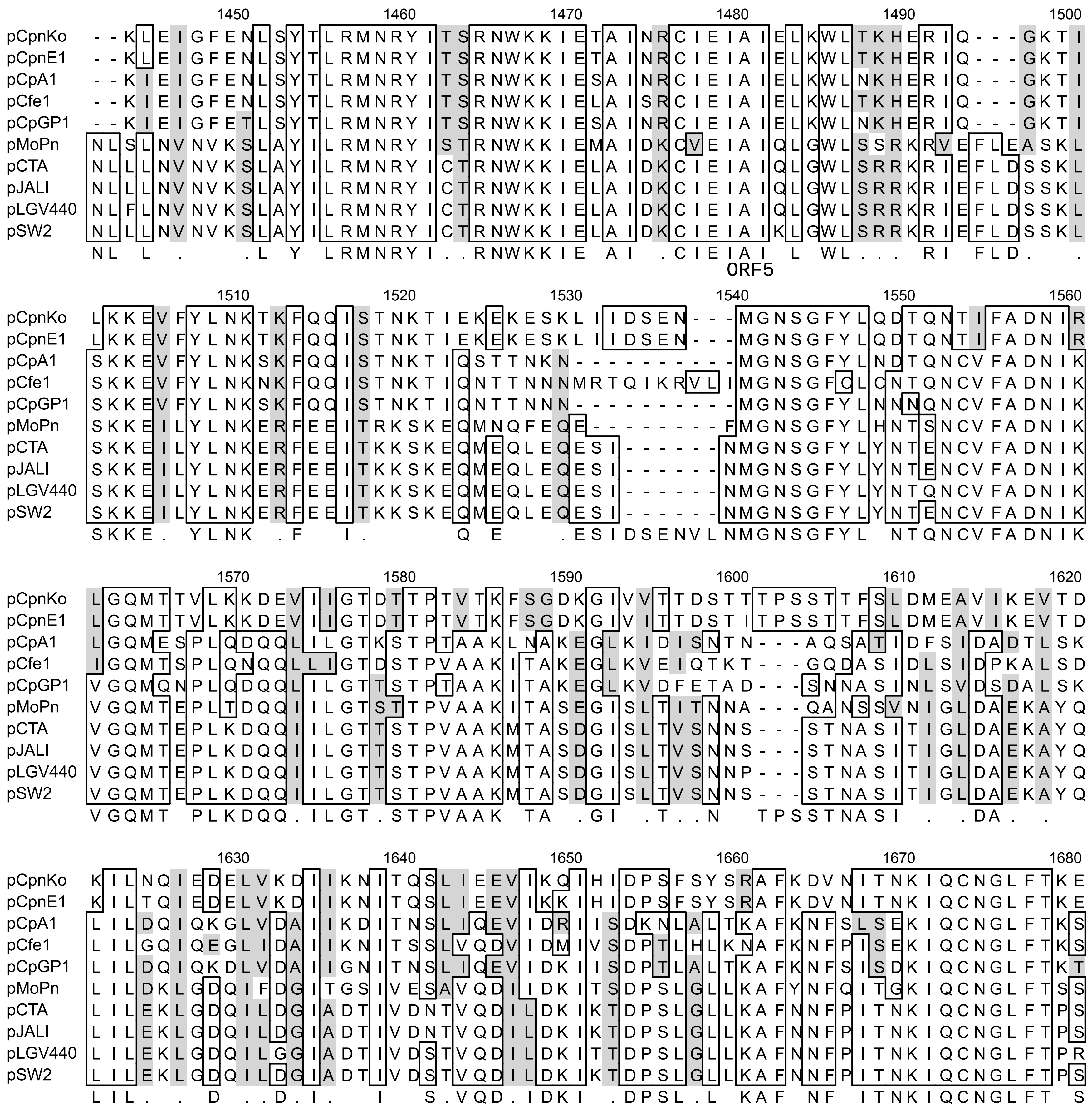
1450 1460 1470 1480 1490 1500
pCpnKo --KLEIGFENLSYTLRMNRYITSRNWKKIETAINRCIEIAIELKWLTKHERIQ---GKTI
pCpnE1 --KLEIGFENLSYTLRMNRYITSRNWKKIETAINRCIEIAIELKWLTKHERIQ---GKTI
pCpA1 --KIEIGFENLSYTLRMNRYITSRNWKKIESAINRCIEIAIELKWLNKHERIQ---GKTI
pCfe1 --KIEIGFENLSYTLRMNRYITSRNWKKIELAISRCIEIAIELKWLTKHERIQ---GKTI
pCpGP1 --KIEIGFETLSYTLRMNRYITSRNWKKIESAINRCIEIAIELKWLNKHERIQ---GKTI
pMoPn NLSLNVNVKSLAYILRMNRYISTRNWKKIEMAIDKCVEIAIQLGWLSSRKRVEFLEASKL
pCTA NLLLNVNVKSLAYILRMNRYICTRNWKKIELAIDKCIEIAIQLGWLSRRKRIEFLDSSKL
pJALI NLLLNVNVKSLAYILRMNRYICTRNWKKIELAIDKCIEIAIQLGWLSRRKRIEFLDSSKL
pLGV440 NLFLNVNVKSLAYILRMNRYICTRNWKKIELAIDKCIEIAIQLGWLSRRKRIEFLDSSKL
pSW2 NLLLNVNVKSLAYILRMNRYICTRNWKKIELAIDKCIEIAIKLGWLSRRKRIEFLDSSKL
NL L . .L Y LRMNRYI ..RNWKKIE AI .CIEIAI L WL ... RI FLD. .
ORF5
1510 1520 1530 1540 1550 1560
pCpnKo LKKEVFYLNKTKFQQISTNKTIEKEKESKLIIDSEN---MGNSGFYLQDTQNTIFADNIR
pCpnE1 LKKEVFYLNKTKFQQISTNKTIEKEKESKLIIDSEN---MGNSGFYLQDTQNTIFADNIR
pCpA1 SKKEVFYLNKSKFQQISTNKTIQSTTNKN----------MGNSGFYLNDTQNCVFADNIK
pCfe1 SKKEVFYLNKNKFQQISTNKTIQNTTNNNMRTQIKRVLIMGNSGFCLCNTQNCVFADNIK
pCpGP1 SKKEVFYLNKSKFQQISTNKTIQNTTNNN----------MGNSGFYLNNNQNCVFADNIK
pMoPn SKKEILYLNKERFEEITRKSKEQMNQFEQE--------FMGNSGFYLHNTSNCVFADNIK
pCTA SKKEILYLNKERFEEITKKSKEQMEQLEQESI------NMGNSGFYLYNTENCVFADNIK
pJALI SKKEILYLNKERFEEITKKSKEQMEQLEQESI------NMGNSGFYLYNTENCVFADNIK
pLGV440 SKKEILYLNKERFEEITKKSKEQMEQLEQESI------NMGNSGFYLYNTQNCVFADNIK
pSW2 SKKEILYLNKERFEEITKKSKEQMEQLEQESI------NMGNSGFYLYNTENCVFADNIK
SKKE. YLNK .F I. Q E .ESIDSENVLNMGNSGFYL NTQNCVFADNIK
1570 1580 1590 1600 1610 1620
pCpnKo LGQMTTVLKKDEVIIGTDTTPTVTKFSGDKGIVVTTDSTTTPSSTTFSLDMEAVIKEVTD
pCpnE1 LGQMTTVLKKDEVIIGTDTTPTVTKFSGDKGIVITTDSTITPSSTTFSLDMEAVIKEVTD
pCpA1 LGQMESPLQDQQLILGTKSTPTAAKLNAKEGLKIDISNTN---AQSATIDFSIDADTLSK
pCfe1 IGQMTSPLQNQQLLIGTDSTPVAAKITAKEGLKVEIQTKT---GQDASIDLSIDPKALSD
pCpGP1 VGQMQNPLQDQQLILGTTSTPTAAKITAKEGLKVDFETAD---SNNASINLSVDSDALSK
pMoPn VGQMTEPLTDQQIILGTSTTPVAAKITASEGISLTITNNA---QANSSVNIGLDAEKAYQ
pCTA VGQMTEPLKDQQIILGTTSTPVAAKMTASDGISLTVSNNS---STNASITIGLDAEKAYQ
pJALI VGQMTEPLKDQQIILGTTSTPVAAKMTASDGISLTVSNNS---STNASITIGLDAEKAYQ
pLGV440 VGQMTEPLKDQQIILGTTSTPVAAKMTASDGISLTVSNNP---STNASITIGLDAEKAYQ
pSW2 VGQMTEPLKDQQIILGTTSTPVAAKMTASDGISLTVSNNS---STNASITIGLDAEKAYQ
VGQMT PLKDQQ.ILGT.STPVAAK TA .GI .T..N TPSSTNASI . .DA. .
1630 1640 1650 1660 1670 1680
pCpnKo KILNQIEDELVKDIIKNITQSLIEEVIKQIHIDPSFSYSRAFKDVNITNKIQCNGLFTKE
pCpnE1 KILTQIEDELVKDIIKNITQSLIEEVIKKIHIDPSFSYSRAFKDVNITNKIQCNGLFTKE
pCpA1 LILDQIQKGLVDAIIKDITNSLIQEVIDRIISDKNLALTKAFKNFSLSEKIQCNGLFTKS
pCfe1 LILGQIQEGLIDAIIKNITSSLVQDVIDMIVSDPTLHLKNAFKNFPISEKIQCNGLFTKS
pCpGP1 LILDQIQKDLVDAIIGNITNSLIQEVIDKIISDPTLALTKAFKNFSISDKIQCNGLFTKT
pMoPn LILDKLGDQIFDGITGSIVESAVQDIIDKITSDPSLGLLKAFYNFQITGKIQCNGLFTSS
pCTA LILEKLGDQILDGIADTIVDNTVQDILDKIKTDPSLGLLKAFNNFPITNKIQCNGLFTPS
pJALI LILEKLGDQILDGIADTIVDNTVQDILDKIKTDPSLGLLKAFNNFPITNKIQCNGLFTPS
pLGV440 LILEKLGDQILGGIADTIVDSTVQDILDKITTDPSLGLLKAFNNFPITNKIQCNGLFTPR
pSW2 LILEKLGDQILDGIADTIVDSTVQDILDKIKTDPSLGLLKAFNNFPITNKIQCNGLFTPS
LIL. .D ..D.I. I S.VQD.IDKI .DPSL.L KAF NF ITNKIQCNGLFT S

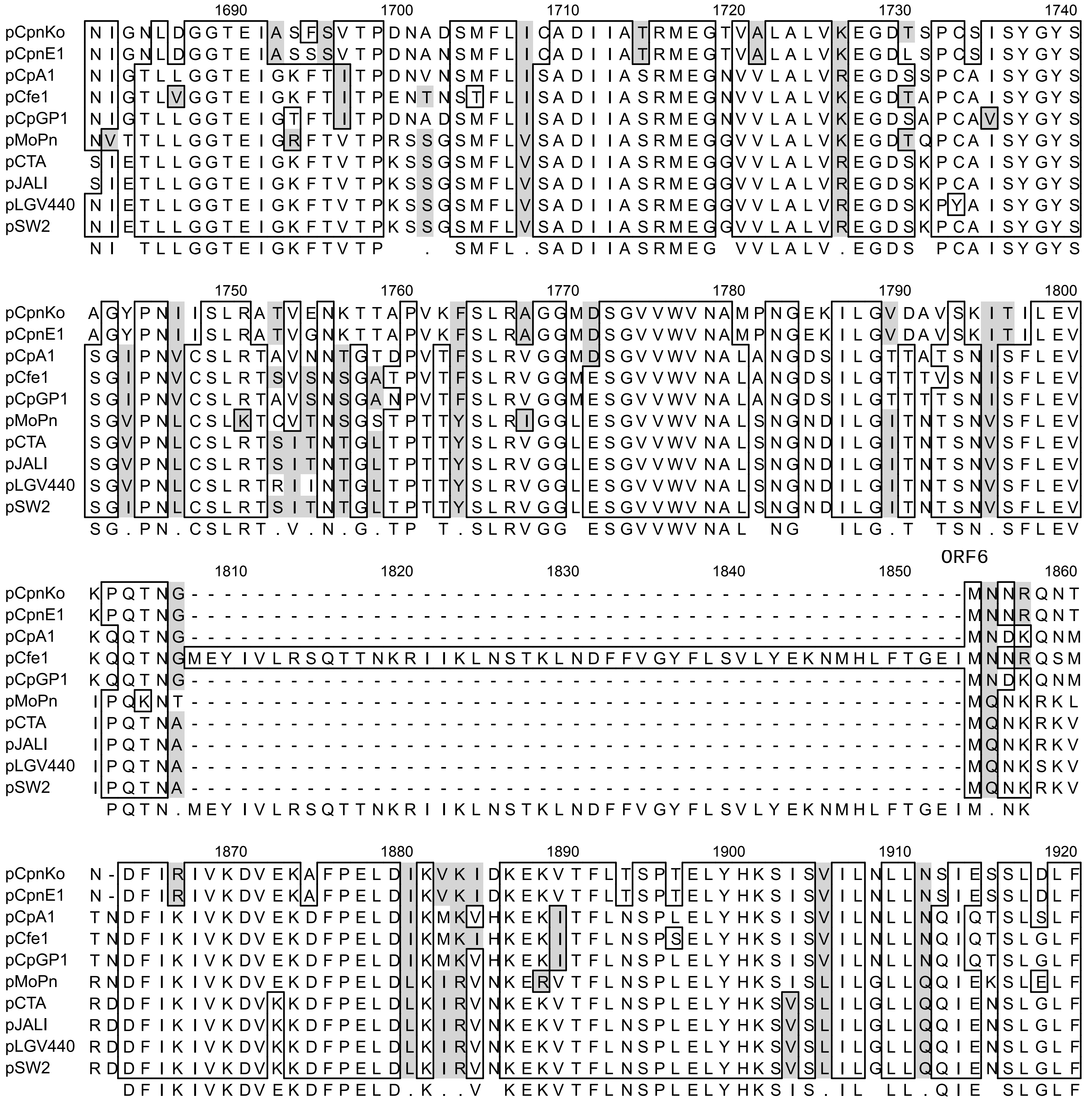

1690 1700 1710 1720 1730 1740
pCpnKo NIGNLDGGTEIASFSVTPDNADSMFLICADIIATRMEGTVALALVKEGDTSPCSISYGYS
pCpnE1 NIGNLDGGTEIASSSVTPDNANSMFLICADIIATRMEGTVALALVKEGDLSPCSISYGYS
pCpA1 NIGTLLGGTEIGKFTITPDNVNSMFLISADIIASRMEGNVVLALVREGDSSPCAISYGYS
pCfe1 NIGTLVGGTEIGKFTITPENTNSTFLISADIIASRMEGNVVLALVKEGDTAPCAISYGYS
pCpGP1 NIGTLLGGTEIGTFTITPDNADSMFLISADIIASRMEGNVVLALVKEGDSAPCAVSYGYS
pMoPn NVTTLLGGTEIGRFTVTPRSSGSMFLVSADIIASRMEGGVVLALVKEGDTQPCAISYGYS
pCTA SIETLLGGTEIGKFTVTPKSSGSMFLVSADIIASRMEGGVVLALVREGDSKPCAISYGYS
pJALI SIETLLGGTEIGKFTVTPKSSGSMFLVSADIIASRMEGGVVLALVREGDSKPCAISYGYS
pLGV440 NIETLLGGTEIGKFTVTPKSSGSMFLVSADIIASRMEGGVVLALVREGDSKPYAISYGYS
pSW2 NIETLLGGTEIGKFTVTPKSSGSMFLVSADIIASRMEGGVVLALVREGDSKPCAISYGYS
NI TLLGGTEIGKFTVTP . SMFL.SADIIASRMEG VVLALV.EGDS PCAISYGYS
1750 1760 1770 1780 1790 1800
pCpnKo AGYPNIISLRATVENKTTAPVKFSLRAGGMDSGVVWVNAMPNGEKILGVDAVSKITILEV
pCpnE1 AGYPNIISLRATVGNKTTAPVKFSLRAGGMDSGVVWVNAMPNGEKILGVDAVSKITILEV
pCpA1 SGIPNVCSLRTAVNNTGTDPVTFSLRVGGMDSGVVWVNALANGDSILGTTATSNISFLEV
pCfe1 SGIPNVCSLRTSVSNSGATPVTFSLRVGGMESGVVWVNALANGDSILGTTTVSNISFLEV
pCpGP1 SGIPNVCSLRTAVSNSGANPVTFSLRVGGMESGVVWVNALANGDSILGTTTTSNISFLEV
pMoPn SGVPNLCSLKTCVTNSGSTPTTYSLRIGGLESGVVWVNALSNGNDILGITNTSNVSFLEV
pCTA SGVPNLCSLRTSITNTGLTPTTYSLRVGGLESGVVWVNALSNGNDILGITNTSNVSFLEV
pJALI SGVPNLCSLRTSITNTGLTPTTYSLRVGGLESGVVWVNALSNGNDILGITNTSNVSFLEV
pLGV440 SGVPNLCSLRTRIINTGLTPTTYSLRVGGLESGVVWVNALSNGNDILGITNTSNVSFLEV
pSW2 SGIPNLCSLRTSITNTGLTPTTYSLRVGGLESGVVWVNALSNGNDILGITNTSNVSFLEV
SG.PN.CSLRT.V.N.G.TP T.SLRVGG ESGVVWVNAL NG ILG.T TSN.SFLEV
ORF6
1810 1820 1830 1840 1850 1860
pCpnKo KPQTNG-------------------------------------MNNRQNT
pCpnE1 KPQTNG-------------------------------------MNNRQNT
pCpA1 KQQTNG-------------------------------------MNDKQNM
pCfe1 KQQTNGMEYIVLRSQTTNKRIIKLNSTKLNDFFVGYFLSVLYEKNMHLFTGEIMNNRQSM
pCpGP1 KQQTNG-------------------------------------MNDKQNM
pMoPn IPQKNT-------------------------------------MQNKRKL
pCTA IPQTNA-------------------------------------MQNKRKV
pJALI IPQTNA-------------------------------------MQNKRKV
pLGV440 IPQTNA-------------------------------------MQNKSKV
pSW2 IPQTNA-------------------------------------MQNKRKV
PQTN.MEYIVLRSQTTNKRIIKLNSTKLNDFFVGYFLSVLYEKNMHLFTGEIM.NK
1870 1880 1890 1900 1910 1920
pCpnKo N-DFIRIVKDVEKAFPELDIKVKIDKEKVTFLTSPTELYHKSISVILNLLNSIESSLDLF
pCpnE1 N-DFIRIVKDVEKAFPELDIKVKIDKEKVTFLTSPTELYHKSISVILNLLNSIESSLDLF
pCpA1 TNDFIKIVKDVEKDFPELDIKMKVHKEKITFLNSPLELYHKSISVILNLLNQIQTSLSLF
pCfe1 TNDFIKIVKDVEKDFPELDIKMKIHKEKITFLNSPSELYHKSISVILNLLNQIQTSLGLF
pCpGP1 TNDFIKIVKDVEKDFPELDIKMKVHKEKITFLNSPLELYHKSISVILNLLNQIQTSLGLF
pMoPn RNDFIKIVKDVEKDFPELDLKIRVNKERVTFLNSPLELYHKSISLILGLLQQIEKSLELF
pCTA RDDFIKIVKDVKKDFPELDLKIRVNKEKVTFLNSPLELYHKSVSLILGLLQQIENSLGLF
pJALI RDDFIKIVKDVKKDFPELDLKIRVNKEKVTFLNSPLELYHKSVSLILGLLQQIENSLGLF
pLGV440 RDDFIKIVKDVKKDFPELDLKIRVNKEKVTFLNSPLELYHKSVSLILGLLQQIENSLGLF
pSW2 RDDFIKIVKDVKKDFPELDLKIRVNKEKVTFLNSPLELYHKSVSLILGLLQQIENSLGLF
DFIKIVKDVEKDFPELD.K..V KEKVTFLNSPLELYHKSIS.IL LL.QIE SLGLF

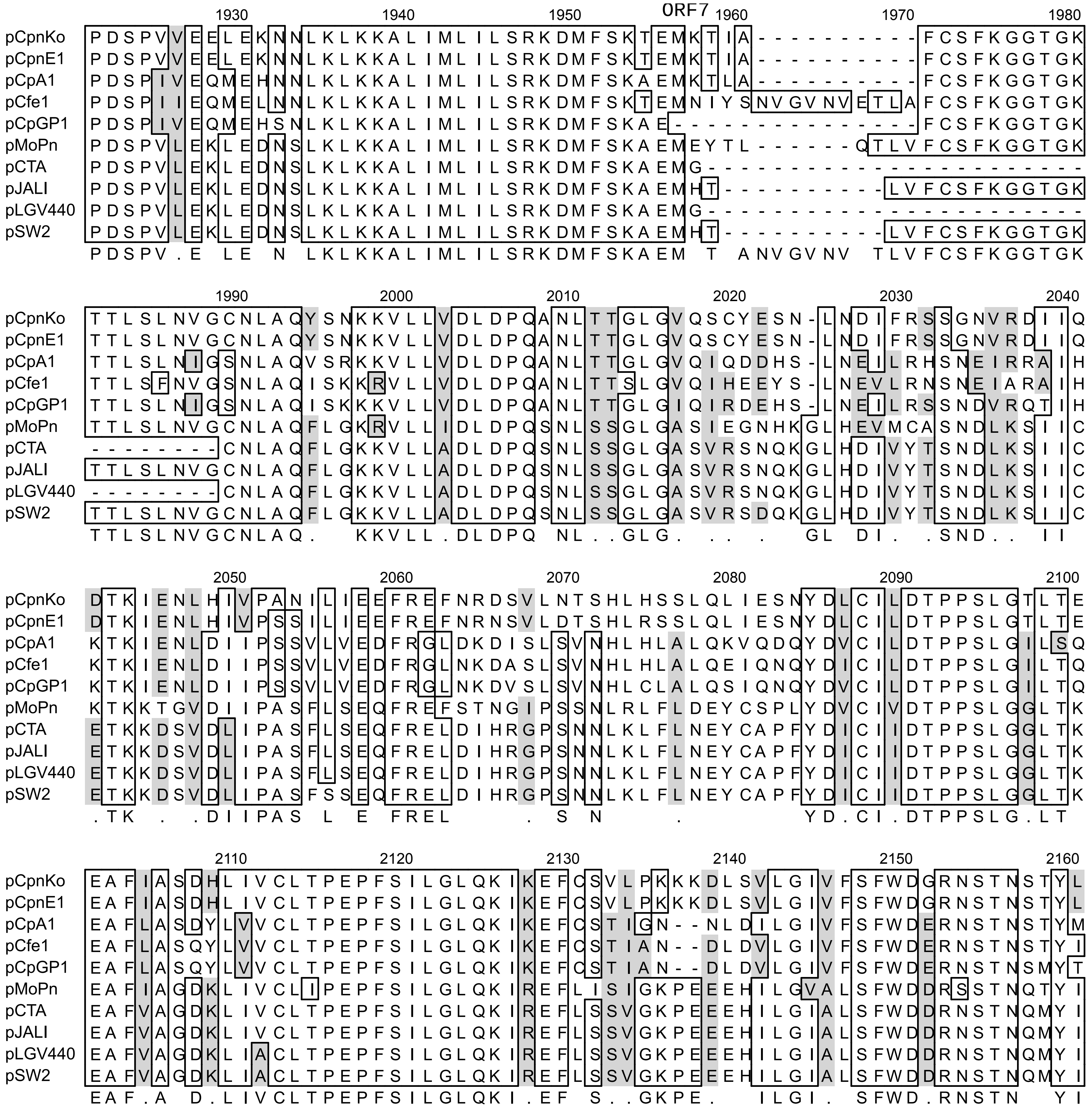

1930 1940 1950 ORF7 1960 1970 1980
pCpnKo PDSPVVEELEKNNLKLKKALIMLILSRKDMFSKTEMKTIA-----------FCSFKGGTGK
pCpnE1 PDSPVVEELEKNNLKLKKALIMLILSRKDMFSKTEMKTIA-----------FCSFKGGTGK
pCpA1 PDSPIVEQMEHNNLKLKKALIMLILSRKDMFSKAEMKTLA-----------FCSFKGGTGK
pCfe1 PDSPIIEQMELNNLKLKKALIMLILSRKDMFSKTEMNIYSNVGVNVETLAFCSFKGGTGK
pCpGP1 PDSPIVEQMEHSNLKLKKALIMLILSRKDMFSKAE---------------FCSFKGGTGK
pMoPn PDSPVLEKLEDNSLKLKKALIMLILSRKDMFSKAEMEYTL------QTLVFCSFKGGTGK
pCTA PDSPVLEKLEDNSLKLKKALIMLILSRKDMFSKAEMG-----------------------
pJALI PDSPVLEKLEDNSLKLKKALIMLILSRKDMFSKAEMHT----------LVFCSFKGGTGK
pLGV440 PDSPVLEKLEDNSLKLKKALIMLILSRKDMFSKAEMG-----------------------
pSW2 PDSPVLEKLEDNSLKLKKALIMLILSRKDMFSKAEMHT----------LVFCSFKGGTGK
PDSPV.E LE N LKLKKALIMLILSRKDMFSKAEM T ANVGVNV TLVFCSFKGGTGK
1990 2000 2010 2020 2030 2040
pCpnKo TTLSLNVGCNLAQYSNKKVLLVDLDPQANLTTGLGVQSCYESN-LNDIFRSSGNVRDIIQ
pCpnE1 TTLSLNVGCNLAQYSNKKVLLVDLDPQANLTTGLGVQSCYESN-LNDIFRSSGNVRDIIQ
pCpA1 TTLSLNIGSNLAQVSRKKVLLVDLDPQANLTTGLGVQIQDDHS-LNEILRHSNEIRRAIH
pCfe1 TTLSFNVGSNLAQISKKRVLLVDLDPQANLTTSLGVQIHEEYS-LNEVLRNSNEIARAIH
pCpGP1 TTLSLNIGSNLAQISKKKVLLVDLDPQANLTTGLGIQIRDEHS-LNEILRSSNDVRQTIH
pMoPn TTLSLNVGCNLAQFLGKRVLLIDLDPQSNLSSGLGASIEGNHKGLHEVMCASNDLKSIIC
pCTA ---------CNLAQFLGKKVLLADLDPQSNLSSGLGASVRSNQKGLHDIVYTSNDLKSIIC
pJALI TTLSLNVGCNLAQFLGKKVLLADLDPQSNLSSGLGASVRSNQKGLHDIVYTSNDLKSIIC
pLGV440 ---------CNLAQFLGKKVLLADLDPQSNLSSGLGASVRSNQKGLHDIVYTSNDLKSIIC
pSW2 TTLSLNVGCNLAQFLGKKVLLADLDPQSNLSSGLGASVRSDQKGLHDIVYTSNDLKSIIC
TTLSLNVGCNLAQ. KKVLL.DLDPQ NL..GLG. . . . GL DI. .SND.. II
2050 2060 2070 2080 2090 2100
pCpnKo DTKIENLHIVPANILIEEFREFNRDSVLNTSHLHSSLQLIESNYDLCILDTPPSLGTLTE
pCpnE1 DTKIENLHIVPSSILIEEFREFNRNSVLDTSHLRSSLQLIESNYDLCILDTPPSLGTLTE
pCpA1 KTKIENLDIIPSSVLVEDFRGLDKDISLSVNHLHLALQKVQDQYDVCILDTPPSLGILSQ
pCfe1 KTKIENLDIIPSSVLVEDFRGLNKDASLSVNHLHLALQEIQNQYDICILDTPPSLGILTQ
pCpGP1 KTKIENLDIIPSSVLVEDFRGLNKDVSLSVNHLCLALQSIQNQYDVCILDTPPSLGILTQ
pMoPn KTKKTGVDIIPASFLSEQFREFSTNGIPSSNLRLFLDEYCSPLYDVCIVDTPPSLGGLTK
pCTA ETKKDSVDLIPASFLSEQFRELDIHRGPSNNLKLFLNEYCAPFYDICIIDTPPSLGGLTK
pJALI ETKKDSVDLIPASFLSEQFRELDIHRGPSNNLKLFLNEYCAPFYDICIIDTPPSLGGLTK
pLGV440 ETKKDSVDLIPASFLSEQFRELDIHRGPSNNLKLFLNEYCAPFYDICIIDTPPSLGGLTK
pSW2 ETKKDSVDLIPASFSSEQFRELDIHRGPSNNLKLFLNEYCAPFYDICIIDTPPSLGGLTK
.TK . .DIIPAS L E FREL . S N . YD.CI.DTPPSLG.LT
2110 2120 2130 2140 2150 2160
pCpnKo EAFIASDHLIVCLTPEPFSILGLQKIKEFCSVLPKKKDLSVLGIVFSFWDGRNSTNSTYL
pCpnE1 EAFIASDHLIVCLTPEPFSILGLQKIKEFCSVLPKKKDLSVLGIVFSFWDGRNSTNSTYL
pCpA1 EAFLASDYLVVCLTPEPFSILGLQKIKEFCSTIGN--NLDILGIVFSFWDERNSTNSTYM
pCfe1 EAFLASQYLVVCLTPEPFSILGLQKIKEFCSTIAN--DLDVLGIVFSFWDERNSTNSTYI
pCpGP1 EAFLASQYLVVCLTPEPFSILGLQKIKEFCSTIAN--DLDVLGIVFSFWDERNSTNSMYT
pMoPn EAFIAGDKLIVCLIPEPFSILGLQKIREFLISIGKPEEEHILGVALSFWDDRSSTNQTYI
pCTA EAFVAGDKLIVCLTPEPFSILGLQKIREFLSSVGKPEEEHILGIALSFWDDRNSTNQMYI
pJALI EAFVAGDKLIVCLTPEPFSILGLQKIREFLSSVGKPEEEHILGIALSFWDDRNSTNQMYI
pLGV440 EAFVAGDKLIACLTPEPFSILGLQKIREFLSSVGKPEEEHILGIALSFWDDRNSTNQMYI
pSW2 EAFVAGDKLIACLTPEPFSILGLQKIREFLSSVGKPEEEHILGIALSFWDDRNSTNQMYI
EAF.A D.LIVCLTPEPFSILGLQKI.EF S..GKPE. ILGI. SFWD.RNSTN YI

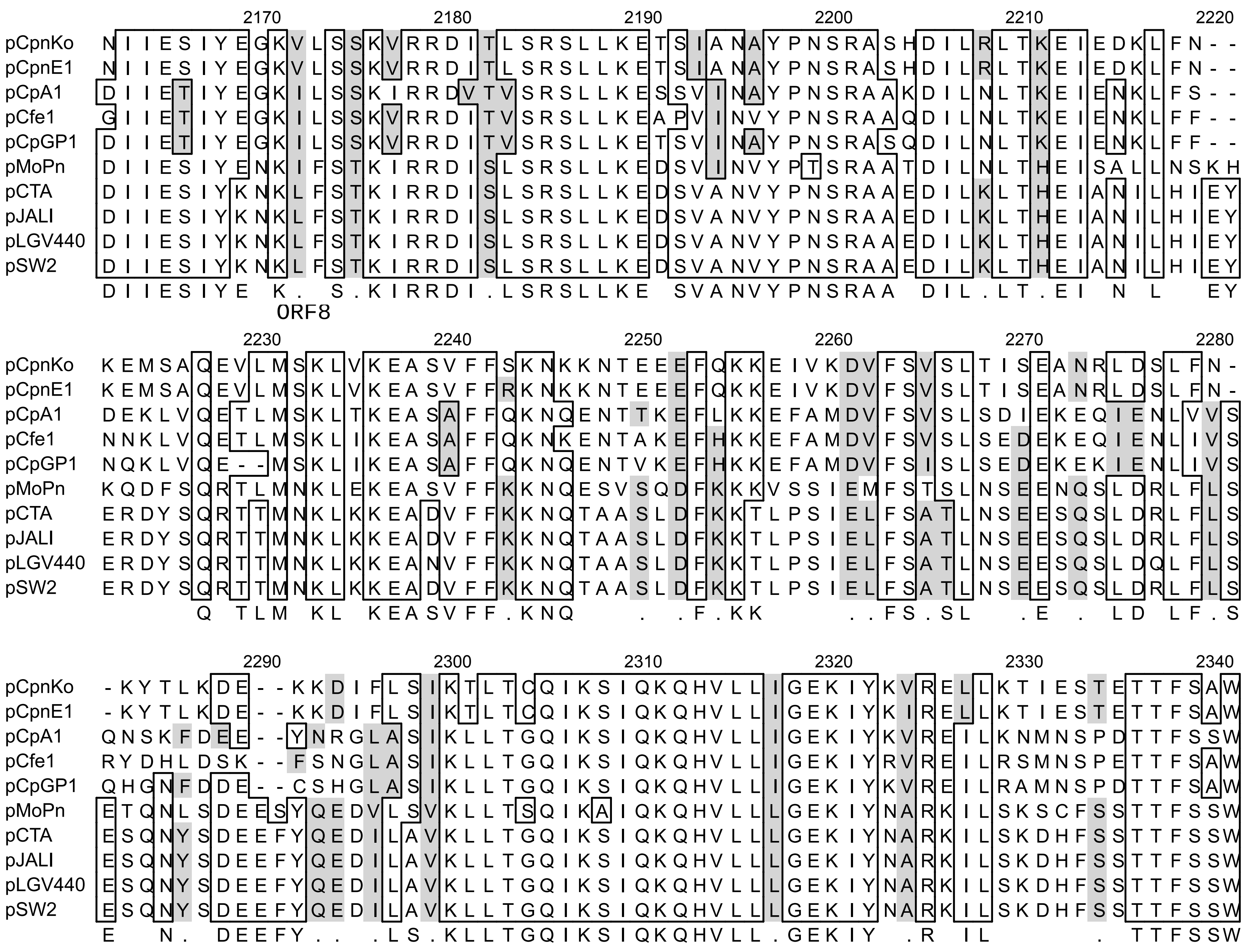
2170 2180 2190 2200 2210 2220
pCpnKo N I I E S I Y E G K V L S S K V R R D I T L S R S L L K E T S I A N A Y P N S R A S H D I L R L T K E I E D K L F N - -
pCpnE1 N I I E S I Y E G K V L S S K V R R D I T L S R S L L K E T S I A N A Y P N S R A S H D I L R L T K E I E D K L F N - -
pCpA1 D I I E T I Y E G K I L S S K I R R D V T V S R S L L K E S S V I N A Y P N S R A A K D I L N L T K E I E N K L F S - -
pCfe1 G I I E T I Y E G K I L S S K V R R D I T V S R S L L K E A P V I N V Y P N S R A A Q D I L N L T K E I E N K L F F - -
pCpGP1 D I I E T I Y E G K I L S S K V R R D I T V S R S L L K E T S V I N A Y P N S R A S Q D I L N L T K E I E N K L F F - -
pMoPn D I I E S I Y E N K I F S T K I R R D I S L S R S L L K E D S V I N V Y P T S R A A T D I L N L T H E I S A L L N S K H
pCTA D I I E S I Y K N K L F S T K I R R D I S L S R S L L K E D S V A N V Y P N S R A A E D I L K L T H E I A N I L H I E Y
pJALI D I I E S I Y K N K L F S T K I R R D I S L S R S L L K E D S V A N V Y P N S R A A E D I L K L T H E I A N I L H I E Y
pLGV440 D I I E S I Y K N K L F S T K I R R D I S L S R S L L K E D S V A N V Y P N S R A A E D I L K L T H E I A N I L H I E Y
pSW2 D I I E S I Y K N K L F S T K I R R D I S L S R S L L K E D S V A N V Y P N S R A A E D I L K L T H E I A N I L H I E Y
D I I E S I Y E K . S . K I R R D I . L S R S L L K E S V A N V Y P N S R A A D I L . L T . E I N L E Y
ORF8
2230 2240 2250 2260 2270 2280
pCpnKo K E M S A Q E V L M S K L V K E A S V F F S K N K K N T E E E F Q K K E I V K D V F S V S L T I S E A N R L D S L F N -
pCpnE1 K E M S A Q E V L M S K L V K E A S V F F R K N K K N T E E E F Q K K E I V K D V F S V S L T I S E A N R L D S L F N -
pCpA1 D E K L V Q E T L M S K L T K E A S A F F Q K N Q E N T T K E F L K K E F A M D V F S V S L S D I E K E Q I E N L V V S
pCfe1 N N K L V Q E T L M S K L I K E A S A F F Q K N K E N T A K E F H K K E F A M D V F S V S L S E D E K E Q I E N L I V S
pCpGP1 N Q K L V Q E - - M S K L I K E A S A F F Q K N Q E N T V K E F H K K E F A M D V F S I S L S E D E K E K I E N L I V S
pMoPn K Q D F S Q R T L M N K L E K E A S V F F K K N Q E S V S Q D F K K K V S S I E M F S T S L N S E E N Q S L D R L F L S
pCTA E R D Y S Q R T T M N K L K K E A D V F F K K N Q T A A S L D F K K T L P S I E L F S A T L N S E E S Q S L D R L F L S
pJALI E R D Y S Q R T T M N K L K K E A D V F F K K N Q T A A S L D F K K T L P S I E L F S A T L N S E E S Q S L D R L F L S
pLGV440 E R D Y S Q R T T M N K L K K E A N V F F K K N Q T A A S L D F K K T L P S I E L F S A T L N S E E S Q S L D Q L F L S
pSW2 E R D Y S Q R T T M N K L K K E A D V F F K K N Q T A A S L D F K K T L P S I E L F S A T L N S E E S Q S L D R L F L S
Q T L M K L K E A S V F F . K N Q . . F . K K . . F S . S L . E . L D L F . S
2290 2300 2310 2320 2330 2340
pCpnKo - K Y T L K D E - - K K D I F L S I K T L T C Q I K S I Q K Q H V L L I G E K I Y K V R E L L K T I E S T E T T F S A W
pCpnE1 - K Y T L K D E - - K K D I F L S I K T L T C Q I K S I Q K Q H V L L I G E K I Y K I R E L L K T I E S T E T T F S A W
pCpA1 Q N S K F D E E - - Y N R G L A S I K L L T G Q I K S I Q K Q H V L L I G E K I Y K V R E I L K N M N S P D T T F S S W
pCfe1 R Y D H L D S K - - F S N G L A S I K L L T G Q I K S I Q K Q H V L L I G E K I Y R V R E I L R S M N S P E T T F S A W
pCpGP1 Q H G N F D D E - - C S H G L A S I K L L T G Q I K S I Q K Q H V L L I G E K I Y K V R E I L R A M N S P D T T F S A W
pMoPn E T Q N L S D E E S Y Q E D V L S V K L L T S Q I K A I Q K Q H V L L L G E K I Y N A R K I L S K S C F S S T T F S S W
pCTA E S Q N Y S D E E F Y Q E D I L A V K L L T G Q I K S I Q K Q H V L L L G E K I Y N A R K I L S K D H F S S T T F S S W
pJALI E S Q N Y S D E E F Y Q E D I L A V K L L T G Q I K S I Q K Q H V L L L G E K I Y N A R K I L S K D H F S S T T F S S W
pLGV440 E S Q N Y S D E E F Y Q E D I L A V K L L T G Q I K S I Q K Q H V L L L G E K I Y N A R K I L S K D H F S S T T F S S W
pSW2 E S Q N Y S D E E F Y Q E D I L A V K L L T G Q I K S I Q K Q H V L L L G E K I Y N A R K I L S K D H F S S T T F S S W
E N . D E E F Y . . . L S . K L L T G Q I K S I Q K Q H V L L . G E K I Y . R I L . T T F S S W

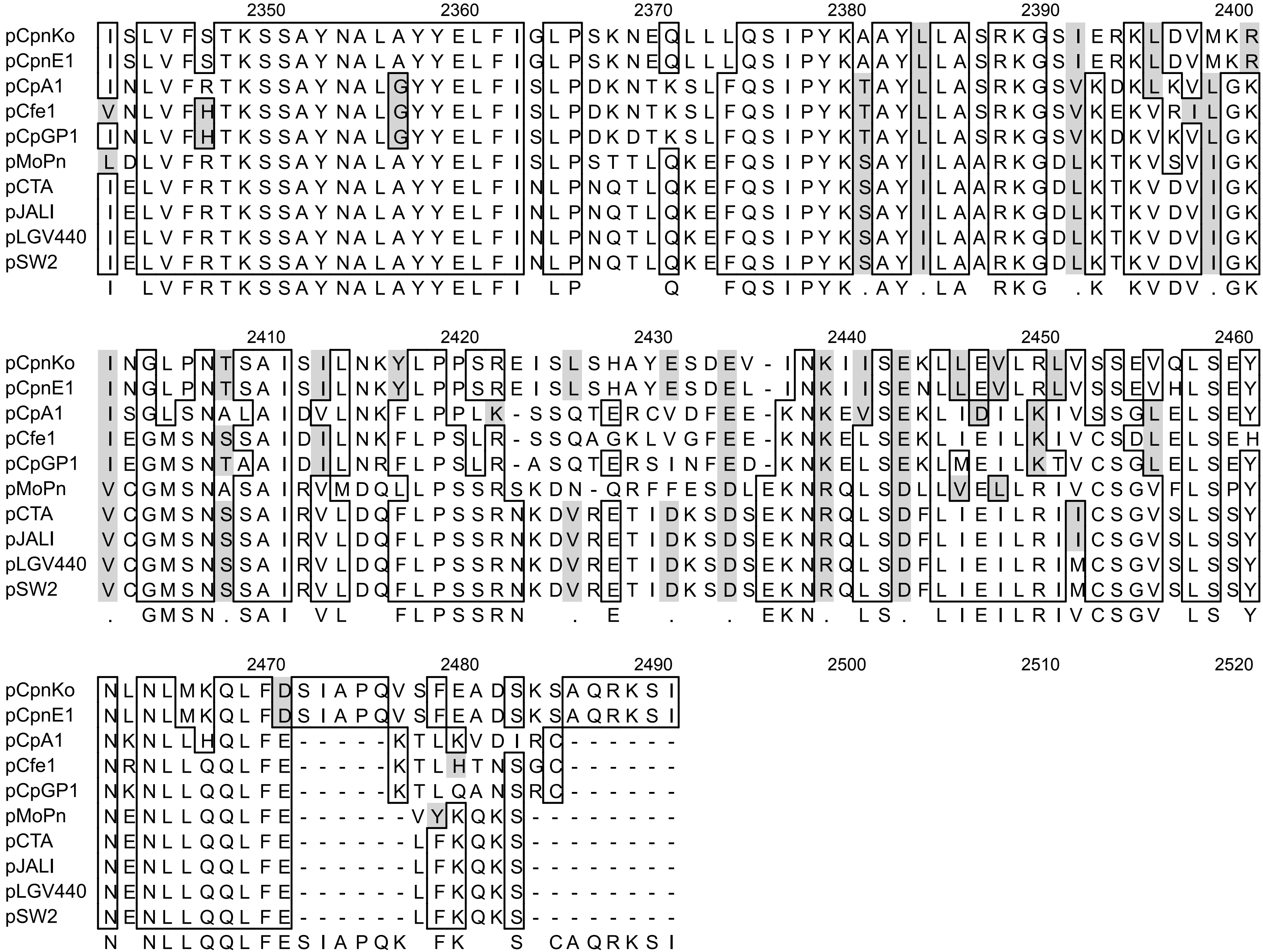

2350 2360 2370 2380 2390 2400
pCpnKo I S L V F S T K S S A Y N A L A Y Y E L F I G L P S K N E Q L L L Q S I P Y K A A Y L L A S R K G S I E R K L D V M K R
pCpnE1 I S L V F S T K S S A Y N A L A Y Y E L F I G L P S K N E Q L L L Q S I P Y K A A Y L L A S R K G S I E R K L D V M K R
pCpA1 I N L V F R T K S S A Y N A L G Y Y E L F I S L P D K N T K S L F Q S I P Y K T A Y L L A S R K G S V K D K L K V L G K
pCfe1 V N L V F H T K S S A Y N A L G Y Y E L F I S L P D K N T K S L F Q S I P Y K T A Y L L A S R K G S V K E K V R I L G K
pCpGP1 I N L V F H T K S S A Y N A L G Y Y E L F I S L P D K D T K S L F Q S I P Y K T A Y L L A S R K G S V K D K V K V L G K
pMoPn L D L V F R T K S S A Y N A L A Y Y E L F I S L P S T T L Q K E F Q S I P Y K S A Y I L A A R K G D L K T K V S V I G K
pCTA I E L V F R T K S S A Y N A L A Y Y E L F I N L P N Q T L Q K E F Q S I P Y K S A Y I L A A R K G D L K T K V D V I G K
pJALI I E L V F R T K S S A Y N A L A Y Y E L F I N L P N Q T L Q K E F Q S I P Y K S A Y I L A A R K G D L K T K V D V I G K
pLGV440 I E L V F R T K S S A Y N A L A Y Y E L F I N L P N Q T L Q K E F Q S I P Y K S A Y I L A A R K G D L K T K V D V I G K
pSW2 I E L V F R T K S S A Y N A L A Y Y E L F I N L P N Q T L Q K E F Q S I P Y K S A Y I L A A R K G D L K T K V D V I G K
I L V F R T K S S A Y N A L A Y Y E L F I L P Q F Q S I P Y K . A Y . L A R K G . K K V D V . G K
2410 2420 2430 2440 2450 2460
pCpnKo I N G L P N T S A I S I L N K Y L P P S R E I S L S H A Y E S D E V - I N K I I S E K L L E V L R L V S S E V Q L S E Y
pCpnE1 I N G L P N T S A I S I L N K Y L P P S R E I S L S H A Y E S D E L - I N K I I S E N L L E V L R L V S S E V H L S E Y
pCpA1 I S G L S N A L A I D V L N K F L P P L K - S S Q T E R C V D F E E - K N K E V S E K L I D I L K I V S S G L E L S E Y
pCfe1 I E G M S N S S A I D I L N K F L P S L R - S S Q A G K L V G F E E - K N K E L S E K L I E I L K I V C S D L E L S E H
pCpGP1 I E G M S N T A A I D I L N R F L P S L R - A S Q T E R S I N F E D - K N K E L S E K L M E I L K T V C S G L E L S E Y
pMoPn V C G M S N A S A I R V M D Q L L P S S R S K D N - Q R F F E S D L E K N R Q L S D L L V E L L R I V C S G V F L S P Y
pCTA V C G M S N S S A I R V L D Q F L P S S R N K D V R E T I D K S D S E K N R Q L S D F L I E I L R I I C S G V S L S S Y
pJALI V C G M S N S S A I R V L D Q F L P S S R N K D V R E T I D K S D S E K N R Q L S D F L I E I L R I I C S G V S L S S Y
pLGV440 V C G M S N S S A I R V L D Q F L P S S R N K D V R E T I D K S D S E K N R Q L S D F L I E I L R I M C S G V S L S S Y
pSW2 V C G M S N S S A I R V L D Q F L P S S R N K D V R E T I D K S D S E K N R Q L S D F L I E I L R I M C S G V S L S S Y
. G M S N . S A I V L F L P S S R N . E . . E K N . L S . L I E I L R I V C S G V L S Y
2470 2480 2490 2500 2510 2520
pCpnKo N L N L M K Q L F D S I A P Q V S F E A D S K S A Q R K S I
pCpnE1 N L N L M K Q L F D S I A P Q V S F E A D S K S A Q R K S I
pCpA1 N K N L L H Q L F E - - - - - K T L K V D I R C - - - - - - -
pCfe1 N R N L L Q Q L F E - - - - - K T L H T N S G C - - - - - - -
pCpGP1 N K N L L Q Q L F E - - - - - K T L Q A N S R C - - - - - - -
pMoPn N E N L L Q Q L F E - - - - - - V Y K Q K S - - - - - - - - -
pCTA N E N L L Q Q L F E - - - - - - L F K Q K S - - - - - - - - -
pJALI N E N L L Q Q L F E - - - - - - L F K Q K S - - - - - - - - -
pLGV440 N E N L L Q Q L F E - - - - - - L F K Q K S - - - - - - - - -
pSW2 N E N L L Q Q L F E - - - - - - L F K Q K S - - - - - - - - -
N N L L Q Q L F E S I A P Q K F K S C A Q R K S I
