# Supplementary material for: Comparison of koala LPCoLN and human strains of Chlamydia pneumoniae highlights extended genetic diversity in the species
Source: BMC Genomics. 2010 Jul 21;11:442. doi: 10.1186/1471-2164-11-442 (PMC3091639; doi:10.1186/1471-2164-11-442)
Supplement: Additional file 10 — Plasmid similarity scores (%). Plasmid similarity scores based on multiple sequence alignment. [file 1471-2164-11-442-S10.DOC]

**Additional file 10**

ClustalW (v1.83) multiple sequence alignment

10 Sequences Aligned Alignment Score = nan

Gaps Inserted = 174 Conserved Identities = 946

Pairwise Alignment Mode: Fast

Pairwise Alignment Parameters:

ktup = 1 Gap Penalty = 3 Top Diagonals = 5 Window Size = 5

Similarity Matrix: gonnet

Multiple Alignment Parameters:

Open Gap Penalty = 10.0 Extend Gap Penalty = 0.1

Delay Divergent = 40% Gap Distance = 8

Similarity Matrix: gonnet

Identity Scores (%)

pCpnKo pCpnE1 pCpA1 pCfe1 pCpGP1 pMoPn pCTA pJALI pLGV440 pSW2

pCpnKo 100.0 96.2 67.7 65.0 68.0 52.0 51.2 51.9 51.2 49.3

pCpnE1 96.8 100.0 65.9 63.0 65.8 50.4 49.5 50.3 49.5 47.6

pCpA1 81.3 78.9 100.0 82.0 88.3 55.9 55.0 55.7 54.9 53.0

pCfe1 77.5 75.1 88.3 100.0 84.4 53.1 52.0 52.8 51.9 50.2

pCpGP1 81.6 79.1 95.0 89.7 100.0 56.0 55.2 55.8 55.2 53.1

pMoPn 69.5 67.7 73.8 69.8 73.1 100.0 84.0 84.9 83.8 80.6

pCTA 68.5 66.8 72.7 69.0 72.9 91.5 100.0 99.0 99.0 93.0

pJALI 69.3 67.5 73.5 69.8 73.5 92.3 99.0 100.0 98.1 93.9

pLGV440 68.3 66.6 72.5 68.8 72.6 91.1 99.3 98.3 100.0 92.6

pSW2 65.6 63.9 69.6 66.1 69.6 87.5 93.2 94.2 92.8 100.0

Similarity Scores (%)
