# Supplementary material for: Comparison of koala LPCoLN and human strains of Chlamydia pneumoniae highlights extended genetic diversity in the species
Source: BMC Genomics. 2010 Jul 21;11:442. doi: 10.1186/1471-2164-11-442 (PMC3091639; doi:10.1186/1471-2164-11-442)
Supplement: Additional file 11 — List of C. pneumoniae target genes. Suggested target genes for detection, strain differentiation and plasmid identification in C. pneumoniae. See also reference [68]. [file 1471-2164-11-442-S11.DOC]

## Additional file 11 - List of *C. pneumoniae* target genes

| **Locus designation^1^** | **Gene length (nt)^2^** | **Predicted or known function and other characteristics** |
| --- | --- | --- |
| **Species-specific genes for detection of *C. pneumoniae*** | | |
| CPK_ORF00391 | 123 | Hypothetical, specific to *C. pneumoniae* koala |
| CPK_ORF00660 | 192 | Hypothetical, specific to *C. pneumoniae* koala |
| CPK_ORF00731 | 117 | Hypothetical, specific to *C. pneumoniae* koala |
| CPK_ORF00969/ CP_0297 | 2073/2097 | Hypothetical |
| CPK_ORF00971/ CP_0295 | 1461/1890 | Hypothetical |
| CPK_ORF00973/ CP_0294 | 2055/695 | Hypothetical |
| CPK_ORF00974/ CP_0293/292/291 | 2034/390/852/798 | Hypothetical |
| CPK_ORF00977/ CP_0290 | 2021/2019 | Hypothetical |
| CPK_ORF00979/ CP_0289/288 | 2028/1170/480 | Hypothetical |
| ***C. pneumoniae* strain differentiation** | | |
| CPK_ORF00237/CP_1042* | 201/558 | Hypothetical, length polymorphism between *C. pneumoniae* human and koala |
| CPK_ORF678* | 966 | Hypothetical, potential *C. pneumoniae* animal-specific gene |
| CPK_ORF00679/CP_0608* | 849/372 | Hypothetical, length polymorphism between *C. pneumoniae* human and koala |
| CPK_ORF00685/CP_0594/593* | 2457/381/1236 | Membrane attack complex / perforin, predicted pathogenic role |
| CPK_ORF00956/CP_0309* | 4218/3831 | Polymorphic membrane protein G6, variable numbers of tandem repeats |
| ***C. pneumoniae* plasmid identification** | | |
| CPK_ORFA00003* | 930 | Site-specific recombinase, phage integrase family, length polymorphism between *C. pneumoniae* koala and horse (N16) |
| CPK_ORFA00005* | 1371 | Replicative DNA helicase (dnaB), unwinding double-stranded DNA |
| CPK_ORFA00007 | 804 | Hypothetical, conserved between *C. pneumoniae* koala and horse (N16) |

**^1^**Koala LPCoLN locus designation (CPK) and human AR39 locus designation (CP).

**^2^**Gene length, relative to koala LPCoLN.

* See Mitchell *et al.* [68] for suggested primer pairs.
